# Interplay between photosynthetic electron flux and organic carbon sinks in sucrose-excreting *Synechocystis* sp. PCC 6803 revealed by omics approaches

**DOI:** 10.1186/s12934-024-02462-6

**Published:** 2024-07-01

**Authors:** Dorota Muth-Pawlak, Lauri Kakko, Pauli Kallio, Eva-Mari Aro

**Affiliations:** Department of Life Technologies, Molecular Plant Biology, University of Turku, Turku, FIN-20014, Finland

## Abstract

**Background:**

Advancing the engineering of photosynthesis-based prokaryotic cell factories is important for sustainable chemical production and requires a deep understanding of the interplay between bioenergetic and metabolic pathways. Rearrangements in photosynthetic electron flow to increase the efficient use of the light energy for carbon fixation must be balanced with a strong carbon sink to avoid photoinhibition. In the cyanobacterium *Synechocystis* sp. PCC 6803, the flavodiiron protein Flv3 functions as an alternative electron acceptor of photosystem I and represents an interesting engineering target for reorganizing electron flow in attempts to enhance photosynthetic CO_2_ fixation and increase production yield.

**Results:**

We have shown that inactivation of Flv3 in engineered sucrose-excreting *Synechocystis* (S02:Δ*flv3*) induces a transition from photoautotrophic sucrose production to mixotrophic growth sustained by sucrose re-uptake and the formation of intracellular carbon sinks such as glycogen and polyhydroxybutyrate. The growth of S02:Δ*flv3* exceeds that of the sucrose-producing strain (S02) and demonstrates unforeseen proteomic and metabolomic changes over the course of the nine-day cultivation. In the absence of Flv3, a down-regulation of proteins related to photosynthetic light reactions and CO_2_ assimilation occurred concomitantly with up-regulation of those related to glycolytic pathways, before any differences in sucrose production between S02 and S02:Δ*flv3* strains were observed. Over time, increased sucrose degradation in S02:Δ*flv3* led to the upregulation of respiratory pathway components, such as the plastoquinone reductase complexes NDH-1_1_ and NDH-2 and the terminal respiratory oxidases Cyd and Cox, which transfer electrons to O_2_. While glycolytic metabolism is significantly up-regulated in S02:Δ*flv3* to provide energy for the cell, the accumulation of intracellular storage compounds and the increase in respiration serve as indirect sinks for photosynthetic electrons.

**Conclusions:**

Our results show that the presence of strong carbon sink in the engineered sucrose-producing *Synechocystis* S02 strain, operating under high light, high CO_2_ and salt stress, cannot compensate for the lack of Flv3 by directly balancing the light transducing source and carbon fixing sink reactions. Instead, the cells immediately sense the imbalance, leading to extensive reprogramming of cellular bioenergetic, metabolic and ion transport pathways that favor mixotrophic growth rather than enhancing photoautotrophic sucrose production.

**Supplementary Information:**

The online version contains supplementary material available at 10.1186/s12934-024-02462-6.

## Introduction

Cyanobacteria are metabolically versatile prokaryotes that have been adapted to different environmental conditions since the beginning of the evolution of water-splitting oxygenic photosynthesis. These organisms are able to flexibly acclimate their metabolism according to the prevailing environment depending on the availability of nutrients, carbon, and light energy. Cells do this by alternating trophic modes, photoautotrophy, photomixotrophy and heterotrophy, to ensure survival and optimal use of available resources. In photoautotrophic growth cyanobacteria rely solely on the use of light energy to drive photosynthetic CO_2_ fixation to produce carbohydrates, which serve as anabolic precursors for all carbon-based organic compounds essential for the cell. Under these conditions, cells also produce and accumulate carbohydrate reserves in the form of glycogen, in addition to other natural storage compounds such as polyhydroxyalkanoates and cyanophycin. Some cyanobacteria, such as the glucose-tolerant laboratory model strain *Synechocystis* sp. PCC 6803 (hereafter *Synechocystis*) [63], can also effectively utilize glucose (Glc) from the extracellular medium, and thereby simultaneously use photosynthesis and glycolysis to grow mixotrophically. Mixotrophy typically gives the cells an advantage as seen in enhanced growth [49] and increased productivity of certain chemical compounds [32, 38, 60].

The transition between metabolic modes requires extensive reorganization of cellular carbon fluxes, including rearrangement of the catabolic and anabolic pathways [36]. In photoautotrophic growth, the cell relies on photosynthetic light reactions to generate energy (ATP) and reducing equivalents (NADPH) to drive carbon fixation in the Calvin-Benson-Bassham (CBB) cycle and the rest of the metabolism. In this mode, the energy balance (ATP/NADPH ratio) of the cell is maintained by electron fluxes between the linear electron transport (LET) chain, the cyclic electron transport (CET) chain and by the availability alternative electron acceptors such as the flavodiiron proteins (Flvs), depending on the availability of light and CO_2_.

The growth conditions and specific electron transfer pathways in use affect the overall cellular energy status and modify the ATP/NADPH ratio according to specific cellular needs, such as photoprotection, carbon fixation or cell growth. Under glycolytic heterotrophic growth, the cell is entirely dependent on carbohydrate catabolism and ultimately on the use of the oxidative tricarboxylic acid (TCA) cycle for the complete oxidation of sugars to CO_2_ [68]. The resulting reducing equivalents are directed to the respiratory chain for ATP synthesis, which regenerates NAD^+^ and allows glycolysis to continue. In this scenario, the relative amount of NADPH is largely determined by the specific catabolic pathways, which in *Synechocystis* include all four major glycolytic pathways found in nature: the Embden–Meyerhof–Parnas (EMP) pathway, the oxidative pentose phosphate (OPP) pathway [17], the phosphoketolase (PKET) [7], pathway and the Entner–Doudoroff (ED) pathway [6]. In addition to providing energy, the catabolic pathways and the TCA cycle provide building blocks for anabolic reactions, which also regulate the relative carbon fluxes according to biosynthetic needs of the cell. In addition to aerobic glycolytic metabolism, *Synechocystis* can cope under anaerobic conditions by using various fermentative pathways to regenerate NAD^+^ when oxygen is not available to run the respiratory chain [54].

In the mixotrophic growth mode, both photoautotrophic and glycolytic metabolism are active. This represents a very complex regulatory challenge for the cyanobacterial cell, as photosynthetic and respiratory electron transfer rely on common enzyme components in a single cellular compartment. The photosynthetic electron transfer chain and the respiratory chain complexes are mainly located in the thylakoid membrane, with the two plastoquinone reductases, NDH-1 and NDH-2 (NdbA in *Synechocystis*) [22, 43] operating at the intersection of these two processes. In the main linear photosynthetic electron transfer pathway, LET, electrons derived from light-driven photosystem II (PSII) water oxidation are directed to the plastoquinone (PQ) pool and then to PSI. After PSI light activation, electrons are transferred via ferredoxin (Fd) and ferredoxin: NADP^+^ oxidoreductase (FNR) to form NADPH, which is then primarily used for CO_2_ fixation in the CBB cycle. Alternatively, if carbon fixation is inhibited, the electrons from Fd can be recycled via NDH-1 to the PQ pool and further either to CET via photosystem I (PSI) or to the respiratory terminal oxidases (RTOs) Cyd/Cox to reduce O_2_. On the other hand, in glycolytic respiration NdbA transfers electrons from NADH [20] produced during carbohydrate degradation, to the PQ pool and further on to the RTOs for O_2_ reduction. To complement these overlapping pathways in cyanobacteria, the Flvs serve as alternative electron valves from photosystems to O_2_.

Flvs are found in all oxygenic photosynthetic organisms, with the exception of flowering plants, and function to protect the photosynthetic apparatus from oxidative damage [1, 65, 67]. *Synechocystis* has four Flv protein isoforms that function primarily as hetero-oligomers Flv1/Flv3 and Flv2/Flv4. These proteins allow excess electrons from LET to be channeled to O_2_ reduction and to form H_2_O, thereby protecting the photosystems when electrons cannot be effectively used for CO_2_ fixation. Based on current knowledge, the electron donor for Flv1/Flv3 is reduced Fd on the acceptor side of PSI [50]. Thus, the electrons from Fd are either allocated to FNR and carbon assimilation, to CET and respiration via NDH-1, or to oxygen photoreduction by Flv1/Flv3. Flv1/3 is the primary pathway for O_2_ photoreduction under high carbon conditions [47], and is particularly important for rapid acclimation during dark-light transitions, as evidenced by its essential role under fluctuating light [1]. This pathway is further synchronized with the flux to RTOs via NDH-1 during the transition between different light and carbon conditions [37], thus serving as an optional pathway to direct excess electrons to O_2_ to protect the photosynthetic system from damage.

In addition to the bioresearch perspective, understanding the interplay between alternative electron transfer pathways and different trophic modes has clear biotechnological implications. While the optimization of culture parameters is a critical part of bioprocess engineering, a deep understanding of the native circuitry of electron transfer reactions is essential for the rational design of efficient production pathways and the development of strains with improved resource allocation. Previous studies have shown that the modulation of alternative electron transfer pathways in *Synechocystis* affects the transition between different trophic modes [56]. Specifically, inactivation of the gene encoding *flv3* (*sll0550)* in the sucrose-producing strain *Synechocystis* S02, was found to induce a transition from the fully photoautotrophic growth mode to the utilization of excreted sugars. Here we have elucidated the molecular-level interplay between the major bioenergetic and metabolic pathways, as well as the major ion transporters, occurring in the engineered sucrose-producing *Synechocystis* strain S02 in the absence of the Flv3 protein (S02:Δ*flv3* strain) over the course of a 9-day cultivation period. The data obtained highlight the urgent need for proteomic and metabolomic approaches to achieve a deep understanding of the source – sink dependency, paving the way for successful design and engineering of photosynthesis-based cyanobacterial cell factories.

## Materials and methods

### Microbial strains

The cyanobacterial strains used in this work are the engineered glucose-tolerant *Synechocystis* sp. PCC 6803 (Kaplan) strains S02 and S02:Δ*flv3* described earlier [56]. The strains overexpress the heterologous sucrose permease (CscB from *E. coli*) and native sucrose phosphate synthase (Sps) in the glucosylglycerol phosphate synthase deletion background (Δ*ggpS*; *sll1566*), and differ from each other only in the absence of the flavodiiron protein Flv3 (Δ*flv3*; *sll0550*) in S02:Δ*flv3*.

### Culture conditions

The strains were grown in liquid BG-11 medium buffered with 20 mM TES-KOH (pH 8.5) [46] supplemented with 20 µg ml^− 1^ spectinomycin (Sp) and 5–8 µg ml^− 1^ chloramphenicol (Cm). The cultivations were carried out in 250 ml Erlenmeyer flasks (medium volume 100 ml) at 30 °C in ~ 120 rpm orbital shaking under 1% CO_2_ atmosphere, using a custom-built LED light system to ensure uniform light conditions in all parallel cultures. The precultures were first grown under 50 µmol photons m^− 2^ s^− 1^ continuous light until OD_750_ ~ 2 and diluted in fresh medium to OD_750_ ~ 0.4. At this point the cultures were divided into four independent replicates, and allowed to acclimate to 400 mM NaCl under 200 µmol photons m^− 2^s ^­1^ for 36 h. After the acclimation period, the main cultures were adjusted to OD_750_ ~ 0.5 by gently pelleting and re-suspending the cells into 100 ml fresh BG-11 containing 400 mM NaCl and 1 mM IPTG, followed by incubation under 200 µmol photons m^− 2^s ^­1^ for 12 days.

### Sampling

Cell samples for monitoring sucrose production and growth were collected from the four parallel cultures at 24 h intervals for 12 consecutive days. For the proteomics and metabolomics analyses, and for quantitating polyhydroxybutarate (PHB) and glycogen, the samples from the four independent cultures of each strain were collected by centrifugation at three time points, 36 h (d1.5), day 5 (d5), and day 9 (d9). All samples were immediately frozen and stored at -80 °C until used for experiments.

### Sample preparation for proteomic analysis

The proteins from the collected 24 samples were isolated and digested using the protocol described previously [22]. The cells were lysed using buffer containing 6 M urea in 0.1 M Tris-NaOH pH 8 buffer with 1% RapiGest (Waters), 1% PMSF, and an equal volume of glass beads in bead beater. The volume of the crude lysate corresponding to 100 µg of protein was then subjected to reduction with DTT, and alkylation with IAA, followed by precipitation of proteins in cold acetone/ethanol mixture in -20 °C overnight. The proteins were then digested with trypsin in 0,05 M Tris -NaOH pH 8 buffer for 20 h. The mixture of peptides was desalted via Sep-Pack C18 (Waters) columns with the protocol recommended by the manufacturer. Immediately before injection, the samples were spiked with iRT synthetic peptides (Biognosys).

### Proteome analysis

The LC-ESI-MS/MS analysis was performed for all the 24 samples on a nanoflow HPLC system (Easy-nLC 2000, Thermo Fisher Scientific) coupled to the Q-Exactive Orbitrap mass spectrometer (Thermo Fisher Scientific) equipped with a nano-electrospray ionization source. The injected samples were first trapped on pre-column and then separated on an analytical C18 column (75 μm x 15 cm, ReproSil-Pur 3 μm 120 Å C18-AQ, Dr. Maisch HPLC GmbH, Ammerbuch-Entringen, Germany) by a two-step, 110 min gradient from 5 to 26% solvent B over 70 min, followed by 26 to 49% B increase over 30 min. The mobile phase consisted of water with 0.1% formic acid (solvent A) or acetonitrile/water (80:20 (v/v)) with 0.1% formic acid (solvent B).

The MS data was acquired automatically by using Thermo Xcalibur 4.1 software (Thermo Fisher Scientific). A data independent acquisition (DIA) method consisted of an Orbitrap MS survey scan of mass range 300–1800 m/z followed by a set of HCD fragmentation spectra with constant tandem mass spectrometry windows with 15-m/z isolation windows covering M/z 400–1000. The spectra were registered with a resolution of 120.000 and 30.000 (at m/z 200) for full scan and for fragment spectra, respectively, and normalized collision energy of 27%. The automatic gain control (AGC) was set to a maximum fill time of 50 ms and maximum number of 3e6 ions for MS scan while for MS2 maximum fill time was set to automatic mode with control of maximum number of 1e6 ions.

Spectral library for identification of peptides from DIA data was combined from 5 gas phase fractionation DDA runs each covering defined m/z range for MS1 precursor scan (300–440)(405–535)(505–635)(600–741)(711–880). MS2 scans were collected in the range of m/z of 200–2000 in all runs. Generated files were then searched together in PD2.5 (Thermo Fisher Scientific) connected to an in-house server running the Mascot 2.6.1 [44] algorithm (Matrix Science), and the local Sequest [11] and Andromeda [8] algorithms were used with precursor and fragment mass tolerance of 10 ppm and 0.02 Da respectively. In addition, trypsin was used as the digestion enzyme, a maximum of 2 missed cleavages were enabled, carbamidomethylation modification was set as static while methionine and N-terminal acetylation modifications as dynamic. The spectra were searched against combined proteome FASTA files from *Synechocystis* sp. PCC6803 [26] (3672 entries, 23.10.2012), *Homo sapiens* (UniProt), *E. coli* (UniProt) and synthetic iRT peptides (Biognosys). For the validation of the spectrum identifications, the Percolator algorithm [24] was used with relaxed false discovery rate (FDR) of 0.05. 1963 proteins were identified including P3000 (CscB) from *E. coli* as well as full set of iRT peptides and these hits were used to generate spectral library and retention time predictor in Skyline software.

Peak picking for the 24 DIA raw files was performed in Skyline with settings allowing the searches of maximum 10 peptides found in spectral library per protein within 4 min time window (+/- 2 min from calculated through iRT predictor). The set of decoy peptides (generated with reverse sequences) was selected to filter out false discovery hits. After manual curation of results, 1837 proteins were detected in Skyline with at least 2 peptides. The coeluting MS2 fragments were integrated, and these values were further processed in MSstats*Shiny* software [30] with linear mixed-effects model algorithm. Three comparisons were performed S02:Δ*flv3* vs. S02 at d1.5, at d5 and at d9 providing estimates of protein fold changes as well as adjusted p values to control FDR at the cutoff 0.05. The original raw data as well as the spectral libraries and Skyline files with all assays used for protein quantification are deposited in Panorama Public data repository [51] associated to ProteomeXchange Consortium [59] under a link https://panoramaweb.org/S02dflv3_timecourse.url (ProteomeXchange ID - PXD050257; DOI 10.6069/9j5y-ws41).

### Sample preparation for metabolome analysis

The 24 collected and OD-normalized samples for metabolite quantitation were shipped in dry ice to the FIMMS Facility at University of Helsinki for analysis. Metabolites were extracted from the cells with the extraction solvent composed of acetonitrile, methanol and water in 40:40:20 ratio, respectively, with 3 cycles of sonication and shaking on vortex. The samples were centrifuged, and the supernatant was evaporated under nitrogen stream, followed by resuspension in 50 µl of extraction solvent immediately prior to injection.

### Metabolome analysis

The quantitative metabolite analysis was carried out using Thermo Vanquish UHPLC + system equipped with SeQuant ZIC-pHILIC column (2.1 × 100 mm, 5-µm particles) coupled to Q-Exactive Orbitrap (Thermo Fisher Scientific) mass spectrometer. Compounds were separated by the gradient elution of 0,1 ml/min using 20mM ammonium carbonate adjusted to pH 9,4 with ammonium solution (25%) as mobile phase A and acetonitrile as mobile phase B. The elution started from 80% B for 2 min with decrease to 20% B for next 17,1 min and maintained until 24 min. The eluent entered in mass spectrometer operating in polarity switching mode with resolution of 35,000 and spray voltages 4250 V for positive and 3250 for negative mode. Instrument control was operated with the Xcalibur 4.1.31.9 software (M/s Thermo Fischer Scientific, Waltham, MA, USA). Peak integration was done with the TraceFinder 4.1 software (M/s Thermo Fischer Scientific, Waltham, MA, USA). The raw data and mz/ml files are available in GNPS-MassIVE data repository [61] with 10.25345/C51834D4P. The peak area data were exported as an Excel file for further analysis. The peak areas for selected metabolites were obtained for specific time points and replicates to calculate fold changes with S02 strain as a control. The statistical significance was calculated with t-test analysis in Perseus software [58].

### Spectrophotometric growth analysis

The growth of the strains was monitored spectrophotometrically by measuring culture optical at 750 nm (OD_750_) using Thermo Scientific Genesys 10 S UV-Vis spectrophotometer. The culture optical densities were routinely calculated from dilutions measured at OD_750_ ~ 0.1–0.5 to ensure readings in the linear area of the spectrophotometer.

### Analysis of sucrose, glycogen and polyhydroxybutyrate

The quantitation of excreted sucrose and the intracellular storage compounds glycogen and PHB were carried out using commercial analytical kits according to the manufacturer’s instructions, but in a smaller scale in 96-well plate format using a microplate reader (Infinite M200 PRO, Tecan, CH). The kits for measuring sucrose (Sucrose/d-Glucose Assay Kit; Megazyme, US) and glycogen (Total Starch Assay Kit; Megazyme, US) were based on spectrophotometric quantitation of d-glucose released from enzymatic breakdown of sucrose in the supernatant (by β-fructosidase) or intracellular glycogen (by α-amylase and amyloglucosidase), respectively, with a coupled glucose oxidase/ peroxidase GOPOD assay (measured at 510 nm). Quantitation of PHB (D-3-Hydroxybutyric Acid Assay Kit; Megazyme, US) was based on alkaline lysis of intracellular PHB into D-3-hydroxybutyric acid, followed by an enzymatic reaction to produce a stoichiometric amount of NADH (by 3-hydroxybutyrate dehydrogenase), coupled to a subsequent NADPH-dependent diaphorase reaction to generate a colored end-product (measured at 492 nm). Commercial standards provided with the kits [57] were used as quantitative reference to make the standard curves for each assay.

## Results

## The growth and sucrose production of the S02 and S02:Δ*flv3* strains

The two engineered sucrose-producing cyanobacterial strains compared in this work were *Synechocystis* S02 and S02:Δ*flv3* [56]. Both strains carry a construct overexpressing the native protein sucrose phosphate synthetase (Sps) and the heterologous transporter sucrose permease (CscB) from *E. coli*, while the endogenous glucosyl glycerol pathway was silenced by the deletion of glucosyl glycerol phosphate synthase gene (*ggps*). The two strains differed from one another only in respect to the *flv3* gene deletion in S02:Δ*flv3*, allowing the comparison of photoautotrophic sucrose production at the proteome and metabolome levels in the presence and the absence of flavodiiron protein Flv3.

**Fig. 1:**
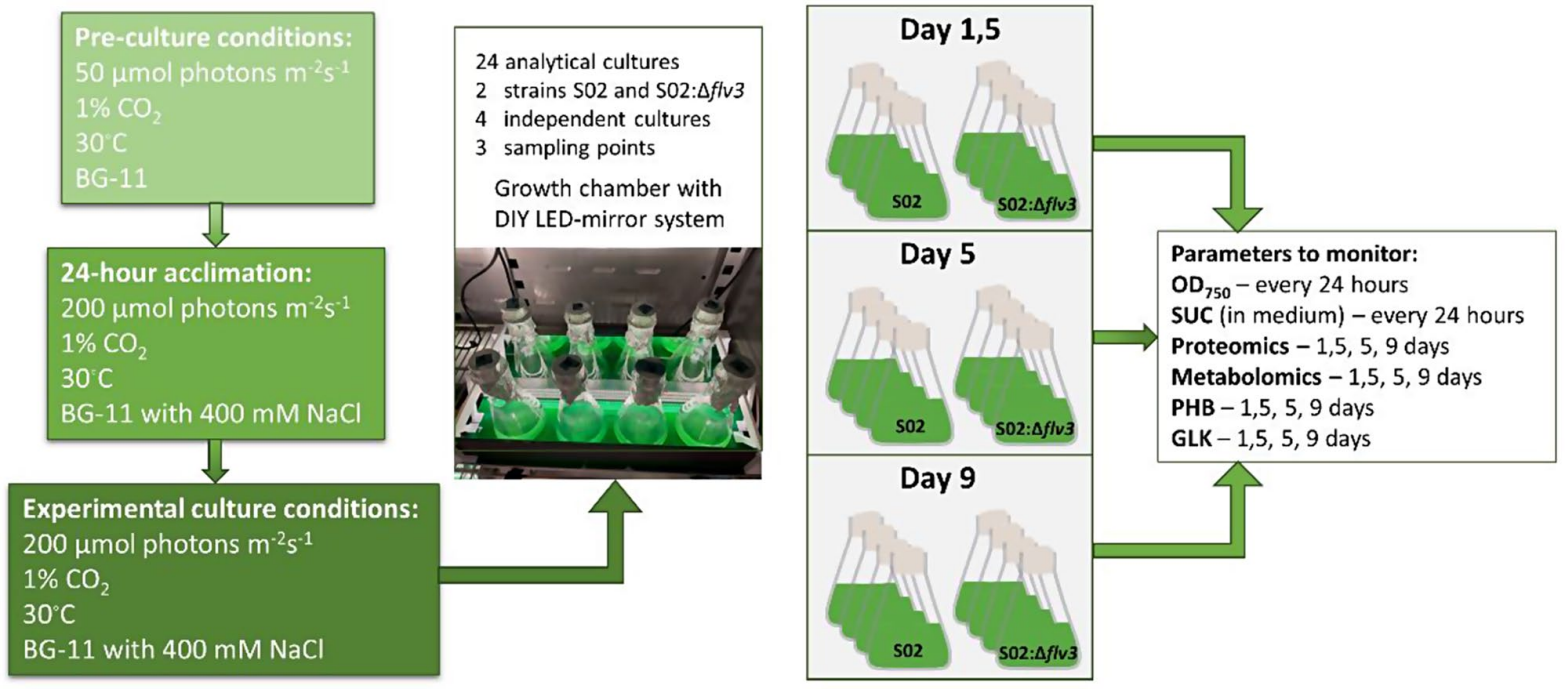
Illustration of the experimental setup. Two *Synechocystis* strains S02 and S02:Δ*flv3* were compared with respect to OD_750_ (optical density measured at 750 nm), sucrose (SUC) production and secretion into the medium, accumulation of polyhydroxybutyrate (PHB) and glycogen (GLK) as well as proteomic and metabolomic changes at three sampling points d1.5, d5 and d9

The data were collected from a single set of cultivation conditions, which were previously shown to result in clear phenotypic differences between the two strains, S02 and S02:Δ*flv3* [56]. In this setup, the cells were exposed to constant osmotic stress (400 mM NaCl) to induce sucrose production and grown under 1% CO_2_ atmosphere and continuous 200 µmol photons m^− 2^s^− 1^ illumination (Fig. 1). The cultivations were carried out as batch cultures in a custom-built growth chamber equipped with LEDs designed to minimize light fluctuations and ensure equal light exposure for all parallel cultures. Both strains were cultured in four independent replicates, and the sampling for cell growth (OD_750_) and sucrose accumulation in the culture medium were performed at 24 h intervals for 12 days. Notably, as all the cultivations were conducted in the presence of 400mM NaCl using engineered ∆*ggps* strains (see Materials and Methods) that lack the native primary response mechanism to osmotic stress (glucosyl glycerol) [35], the setup does not provide conclusive information on the salt stress acclimation of the cells per se, which has been studied at proteome level elsewhere [14, 21, 27].

**Fig. 2:**
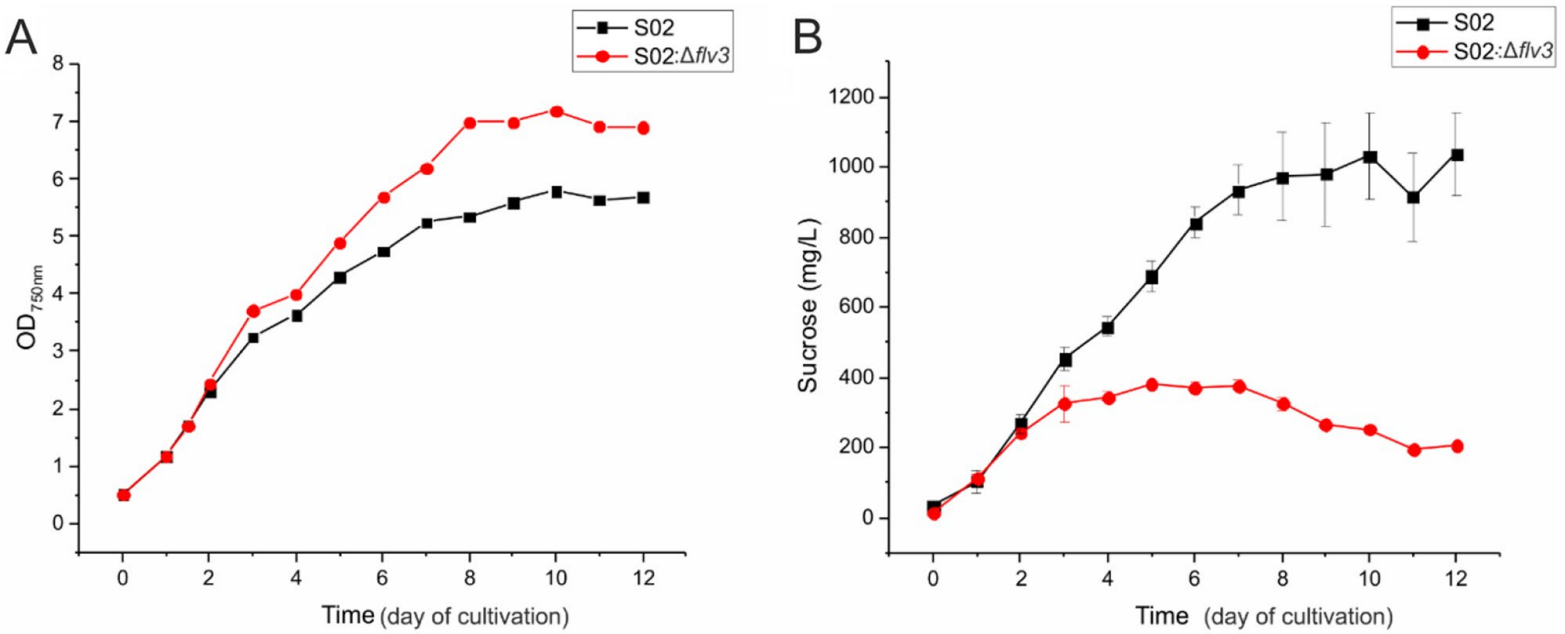
Growth and sucrose production of S02 and S02:Δ*flv3* strains. Comparison of *Synechocystis* strains S02 (black) and S02:Δ*flv3* (red) with respect to growth (**A**) and sucrose production (**B**) monitored daily over a period of 12 days. The mean values and standard deviations for the sucrose production were calculated based on four technical replicates (*N* = 4)

Analysis of cell growth (Fig. 2A) and sucrose productivity (Fig. 2B) revealed clear differences between the strains S02 and S02:Δ*flv3*. After the first two days of cultivation during which the strains behaved in an identical manner, the S02:Δ*flv3* strain grew slightly faster and to a higher density (max. OD_750_ 7 by day 8) in comparison to the S02 strain expressing the native Flv3 (max. OD_750_ 5,5 by day 10) (Fig. 2A). At the same time, the deletion strain S02:Δ*flv3* accumulated significantly less sucrose in the growth medium (max. 400mgL^− 1^ by day 5) than the S02 strain (max. 1000mgL^− 1^ by day 10) (Fig. 2B). Importantly, the overall sucrose profiles were highly reproducible and consistent with the previously published data [56] while some variation was observed in the strain growth patterns, and the absolute sucrose concentrations between the studies. The differences were most likely caused by distinct wavelength profiles between the halogen lamps used earlier and the LEDs in the current setup, but ultimately have no effect on the conclusions of the work.

In order to gain information about metabolic determinants behind the differential behavior of the strains S02 and S02:Δ*flv3*, samples were collected at three different time points based on the growth (Fig. 2A) and sucrose productivity (Fig. 2B) for parallel proteomic and metabolomic analyses (Fig. 1). The first sample was collected on day 1.5 (d1.5) before any changes between strains were observed. The second sample was collected on day 5 (d5) when the growth of S02 started to slow down and the sucrose accumulation for S02:Δ*flv3* had reached the plateau. The third sample was collected on day 9 (d9) when the S02:Δ*flv3* strain was steadily consuming sucrose from the medium, while the S02 sucrose levels had reached the maximum.

## Global proteome differences observed between the S02 and S02:Δ*flv3* strains

**Fig. 3:**
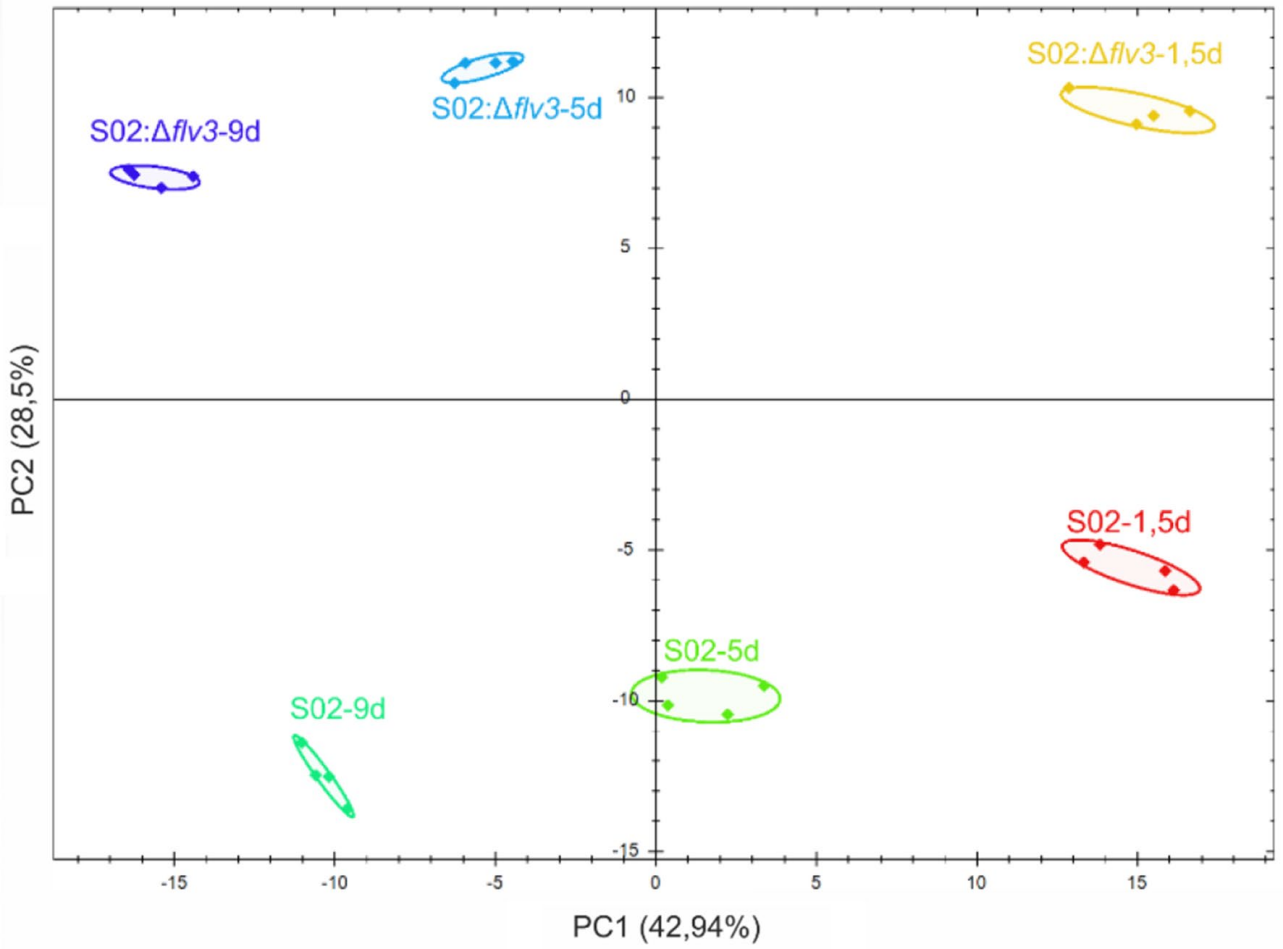
Principal component analysis (PCA) of the proteomic LC-MS/MS data. The data were registered for 4 independent cultures of S02 and S02:Δ*flv3* at three sampling times d1.5, d5 and d9. S02-1.5d in red, S02:Δ*flv3-*1.5d in yellow, S02-5d in green, S02:Δ*flv3-*5d in blue, S02-9d in dark green, S02:Δ*flv3-*9d in violet

**Fig. 4:**
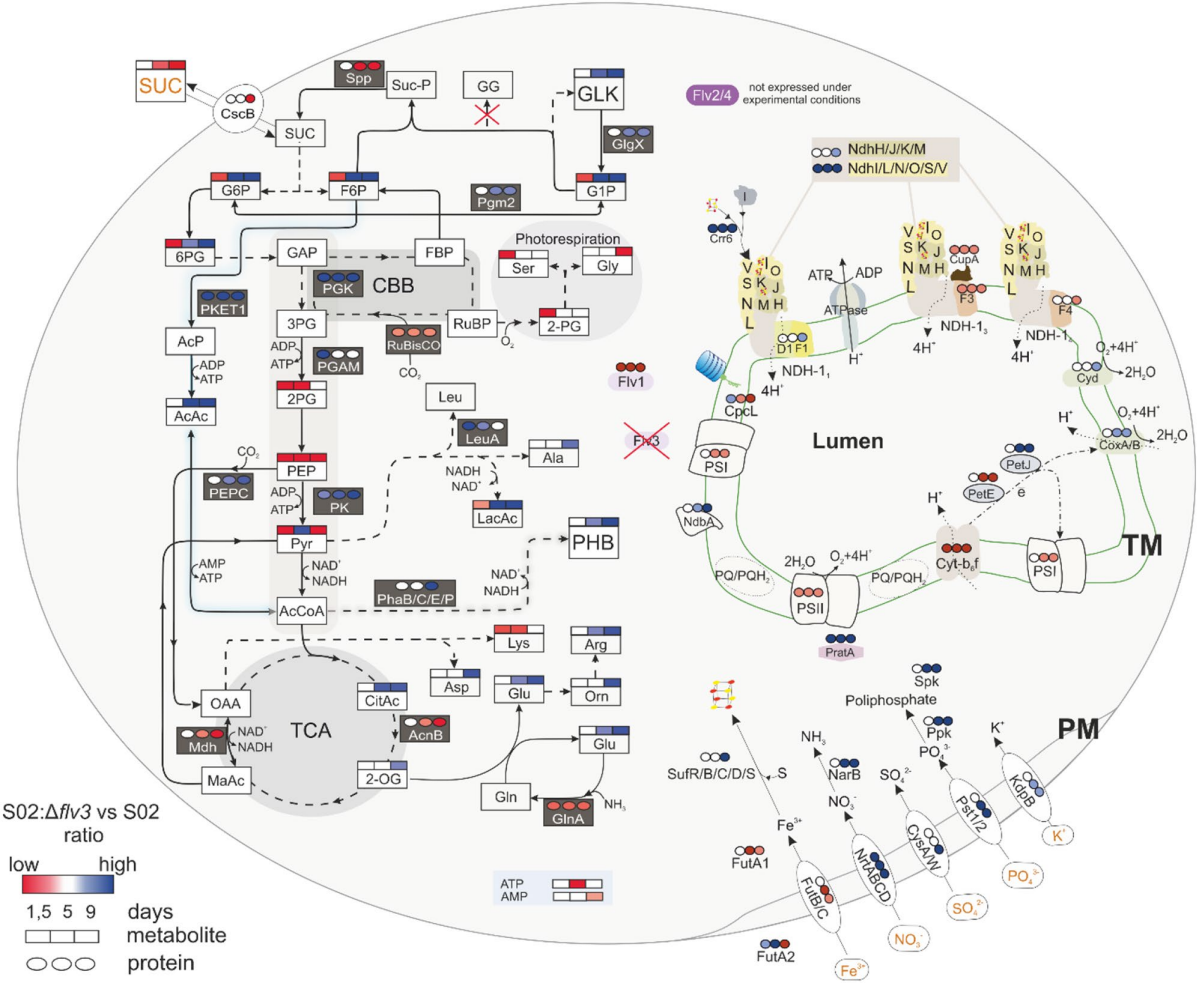
Scheme showing the differential expression of proteins and accumulation of metabolites detected in the strain S02:Δ*flv3* compared to S02 during the sucrose production experiment. Differential proteins expression (circles) and differential metabolite accumulation (squares) are presented above the name of the protein or metabolite reflecting the degree of up- (blue) or down-regulation (red) at consecutive sampling times day 1.5, 5 and 9. The intensity of the color corelates to the level of S02:Δ*flv3* vs. S02 ratio of particular protein. Quantitative data are shown only for proteins and metabolites that meet the statistical threshold of p-value < 0.05 and the practical threshold of -0.58 ≥ log_2_FC ≥ 0.58. White squares and circles represent the values out of the statistical and practical thresholds. Metabolites without squares above names were not measured or no significant difference in their abundance at particular sampling times was detected. Orange text indicates extracellular molecules. A solid arrow indicates a one-step reaction whereas a dashed arrow indicates a reaction involving two or more reaction steps. Proton transport is indicated by dotted lines and electron transport with dash-dotted line. Abbreviations: Spp - sucrose phosphate phosphorylase; CscB - transporter sucrose permease; GG - glucosyl-glycerol; GLK - glycogen; PHB - polihydroxybutarate; Suc-P-sucrose phosphate; SUC-sucrose; G6P - glucose-6-phosphate; G1P - glucose-1-phosphate; F6P - fructose-6-phosphate ; GlgX - glycogen debranching enzyme; PGM2 - phosphoglucomutase; 6PG − 6-phospho-gluconate; GAP - glyceraldehyde-3-phosphate (or G-3-P); FBP - fructose-1,6-bisphosphate; RuBP - ribulose-1,5-bisphosphate; 3PG − 3-phosphoglycerate (or P3G); 2PG − 2-phosphoglycerate (or P2G); PEP - phosphoenolpyruvate; Pyr - pyruvate; AcCoA - acetyl coenzyme A; 2-PG − 2-phosphoglycolate; Ser - serine; Gly - glycine; RuBisCO - ribulose-1,5-bisphosphate carboxylase/oxygenase; CBB - Calvin-Benson-Bassham cycle; PGK -phosphoglycerate kinase; PGAM − 2,3-bisphosphoglycerate-independent phosphoglycerate mutase; PK - pyruvate kinase; PKET1 - phosphoketolase; PEPC - phosphoenolpyruvate carboxylase; AcnB - aconiate hydratase; Mdh - malate dehydrogenase; AcP - acetylphosphate; AcAc - acetatic acid; OAA - oxaloacetate; MaAc - malic acid; CitAc - (iso)citric acid; 2-OG − 2-oxoglutaric acid; LacAc - lactic acid; Glu - glutamic acid; Gln - glutamine; Arg - arginine; Orn - ornithine; Lys - lysine; PSII - photosystem II; Cyt*b*_*6*_*f* -cytochrome *b*_*6*_*f* ; PSI - photosystem I; PratA - tetracopeptide repeat (TPR) protein; CoxA/B – cytochrome c oxidase; Cyd - quinol oxidase; NdbA - NDH-2 protein; PetE - plastocyanin; PetJ - cytochrome c6; CpcL - rod-membrane linker protein; PQ pool - plastoquinone pool; Ccr6 - chlororespiratory reduction 6; CysA/W - sulfate transporters; KdpB - potassium ion transporter; NRT - ABC-type nitrate and nitrite bispecific transporter; NarB - nitrate reductases; SufR/B/C/D/S-iron sulfur cluster biogenesis proteins; GlnA-glutamate-amonia ligase; LeuA − 2-isopropylmalate synthase; SphX- periplasmic phosphate binding protein; Ppk - poliphosphate kinase; PhaP - phasin; PhaB - acetoacetyl-CoA reductase; PhaC/E - heterodimeric PHB synthase; TCA - tricarboxylic acid; Flv (1–4) - flavodiiron proteins

The two sucrose-producing *Synechocystis* strains S02 and S02:Δ*flv3* were subjected to proteome profiling to understand the protein-level changes associated with the metabolic and bioenergetic shifts resulting from the absence of Flv3. The total proteins (membrane and soluble) were extracted from unfractionated cell lysates, and analyzed in quadruplicate with DIA label-free quantification method. The protein abundances were compared between the S02:Δ*flv3* and S02 strains and the results expressed as a S02:Δ*flv3/*S02 ratio in log_2_FC values at the three sampling times: d1.5, d5 and d9 (Supporting Tables 1, 2, 3 and 4). Statistical significance threshold was set to p-value (adj. p-value) ≤ 0.05 while the practical threshold was set to -0.58 ≥ log_2_FC ≥ 0.58 to ensure statistical power ≥ 0,9 (Supporting Fig. 1). As the outcome, the quantitative analysis covered 60% of the *Synechocystis* proteome with at least two peptides per protein for both strains at the three different sampling times. Altogether, DIA allowed the quantification of 1822 proteins, including 38% of membrane proteins theoretically present in *Synechocystis* proteome (Supplemental Table 4). Principal Component Analysis (PCA), performed on quantitative protein data, resulted in clear differentiation of samples representing the two strains and every time point in separate group, which indicated reliable reproducibility of the performed analyses (Fig. 3). In addition, the PCA resolution pattern indicated that the changes in proteomes were induced during the growth period (Supporting Tables 5, 6, 7 and 8) as well as due to the deletion of *flv3* (Supporting Tables 1, 2, 3 and 4). We focused the investigation on differences enhanced by the absence of Flv3 in order to deepen understanding on the underlying mechanisms leading S02:Δ*flv3* to produce less sucrose than S02 (Fig. 4).

### Expression of the engineered target proteins S02 and S02:Δ*flv3*

Based on the analysis, the introduced heterologous sucrose permease (CscB from *E. coli*) and the overexpressed endogenous sucrose phosphate synthase (Sps) were both detected in the engineered strains S02 and S02:Δ*flv3*, with similar relative levels at all time points for Sps and downregulation of CscB in S02:Δ*flv3* on the day 9 of cultivation. Importantly, the analysis also verified that Flv3 was not present in the S02:Δ*flv3* strain, as expected, and no peptides from Flv1, Flv2 or Flv4 were detected. Ggps peptides were not detected in any of the samples confirming that the glucosyl glycerol pathway was inactivated in both strains.

### Differential expression of endogenous proteins in S02 and S02:Δ*flv3* in the course of sucrose production

#### Complexes and individual proteins of linear electron transfer chain

**Fig. 5:**
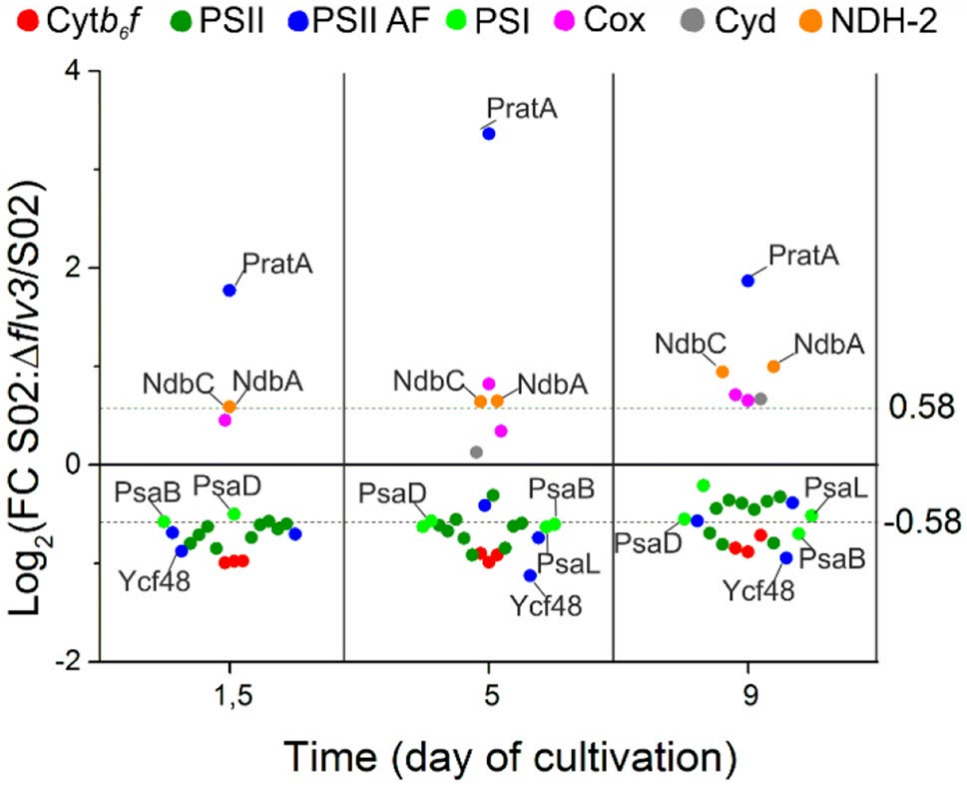
Differential expression of the protein subunits of photosynthetic complexes, associated assembly factors (AF) and respiratory proteins. The values are expressed as log_2_FC of protein abundance in strain S02:Δ*flv3* compared to S02. The data presented are statistically significant with p-value ≤ 0.05. The practical threshold for data interpretation was set at -0.58 ≥ log_2_FC ≥ 0.58.

**Fig. 6:**
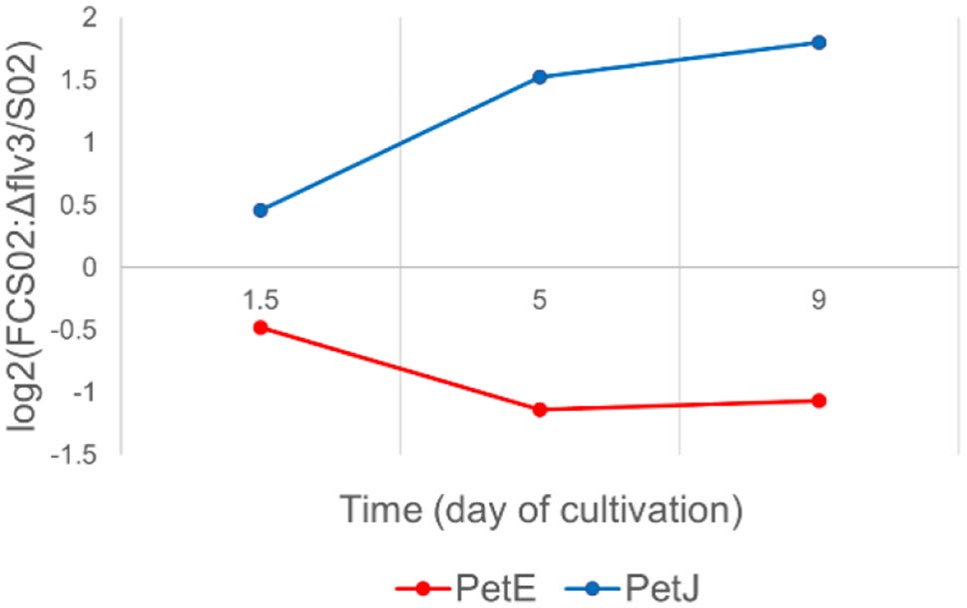
Differential expression of soluble electron carriers PetE and PetJ. The values are expressed as log_2_FC of protein abundance in strain S02:Δ*flv3* compared to S02. The data presented are statistically significant (p-value ≤ 0.05). The practical threshold for data interpretation was set at -0.58 ≥ log_2_FC ≥ 0.58.

Proteomic comparison of the *Synechocystis* strains S02 and S02:Δ*flv3* revealed that *flv3* deletion resulted in coordinated decrease in abundance of photosystem II (PSII) and cytochrome *b*_*6*_*f* (Cyt*b*_*6*_*f*) subunits (Figs. 4 and 5) under the culture conditions. As for PSII, the PsbA2, PsbA3, PsbB(CP47), PsbD(CP43), PsbE, PsbP, PsbV, Psb27, Psb32 and Psb34 decreased in S02:Δ*flv3*. Similarly, the subunits of Cyt*b*_*6*_*f* (PetA, PetB, PetC1) were downregulated in S02:Δ*flv3* strain compared to S02. Several PSI subunits (PsaL, PsaB, PsaD, PsaK2) showed a slight drop in abundance, although to a lower extent than the above-mentioned PSII subunits. An important observation was the increase in abundance of the PSII assembly factor (AF) tetracopeptide repeat (TPR) protein PratA while Ycf48 showed downregulation in S02:Δ*flv3*. The respiratory proteins, CoxA/B and Cyd showed upregulation in S02:Δ*flv3* from d5 to d9 of the experiment. Also NdbA, located in the thylakoid membrane and NdbC located in the plasma membrane, showed increase in abundance on d5 and d9 in S02:Δ*flv3*. The most striking difference between the two strains with respect to soluble electron carriers (SEC) was the substitution of the soluble electron carrier between Cyt*b*_*6*_*f* and PSI from plastocyanin (PetE) to cytochrome c6 (PetJ) from d1.5 to d9 (Fig. 6). The principal photosynthetic ferredoxin in *Synechocystis*, ferredoxin 1 (Fed1) that accepts electrons from PSI, decreased in abundance in S02:Δ*flv3* after 9 days of cultivation. In addition, ferredoxin-NADP^+^ reductase (FNR), transferring electrons from Fed1 to NADP^+^, was downregulated at d1.5 and d5.

#### Differential expression of phycobilisome light-harvesting and linker proteins

**Fig. 7:**
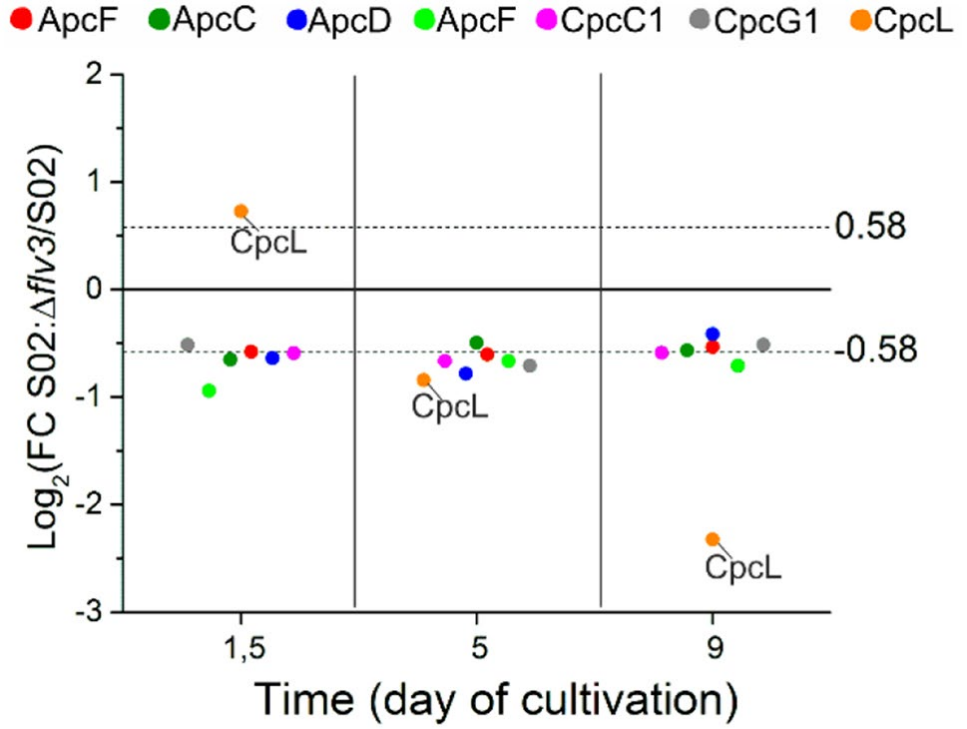
Differential expression of phycobilisome (PBS) protein components. The values are expressed as log_2_FC of protein abundance in strain S02:Δ*flv3* compared to S02. The data presented are statistically significant with p-value ≤ 0.05. The practical threshold for data interpretation was set at -0.58 ≥ log_2_ FC ≥ 0.58.

Consistent with the changes observed for the photosynthetic electron transfer complexes, also the light harvesting structures, phycobilisomes (PBS) were altered in the absence of Flv3. This is seen in the downregulation of allophycocyanin (Apc) proteins (ApcB-F) and linker proteins CpcC1 and CpcG1, the structural components of the PBS core that binds PBS to the PSI or PSII complex, in S02:Δ*flv3* (Fig. 7). Another PSI-associated linker protein CpcL, connecting PBS rod to the thylakoid membrane [70] changed from highly abundant in S02:Δ*flv3* samples at the beginning of the experiment on d1.5 to very low by d5 and d9 (Figs. 4 and 7). The CpcL linker protein is crucial for building the PSI-CpcL-NDH-1 supercomplex that enables cyclic electron transfer in *Synechocystis* [15].

#### Differential expression of the NDH-1 complexes

**Fig. 8:**
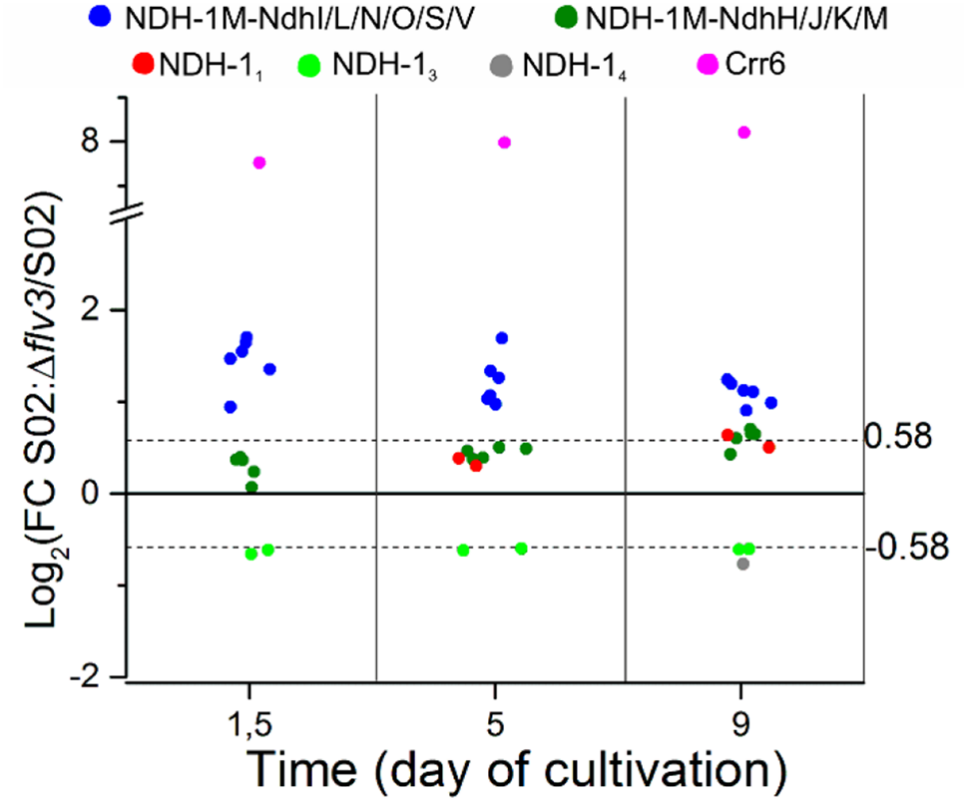
Differential expression of the subunits of the NDH-1 complexes and the associated assembly factor Crr6. The values are expressed as log_2_FC of protein abundance in the strain S02:Δ*flv3* vs. S02. The presented data are statistically significant with p-value ≤ 0.05. The practical threshold for data interpretation was set at -0.58 ≥ log_2_FC ≥ 0.58

*Synechocystis* has four NDH-1 complexes (NDH-1_1-4_) with distinctly different but partially overlapping physiological functions [66]. They all accept electrons from ferredoxin (Fd) and pump protons through the thylakoid membrane to increase the proton motive force (PMF) and ultimately donate the electrons to the PQ pool, thus acting as Fd-PQ oxidoreductases. The four complexes contain a common NDH-1 M core but differ with respect to specific membrane-embedded protein components, with NdhD1 and NdhF1 in NDH-1_1_, NdhD2 and NdhF1 in NDH-1_2_, NdhD3 and NdhF3 with extrinsic CupA and CupS proteins in NDH-1_3_ and finally the NdhD4, NdhF4 and CupB proteins in NDH-1_4_. Considering the abundance of specific NDH-1 complexes during the sucrose-producing experiment (Figs. 4 and 8), the decrease in NdhF3 and CupA as well as in NdhF4 in S02:Δ*flv3* strain indicated decreased amounts of CO_2_ uptake via the inducible NDH-1_3_ and constitutively expressed NDH-1_4_ complexes, respectively. On the other hand, upregulation of NdhD1 and NdhF1 proteins indicated increased cyclic electron transport and/or respiratory activity of the NDH-1_1_ complex in S02:Δ*flv3*. The core subunits of NDH-1 M comprise hydrophilic (NdhH-K, O,S) and hydrophobic (NdhA-C, E,G, L-N) subunits. In the current work, 14 subunits were detected and showed non-stoichiometric differential expression. An increased abundance was detected for NdhI, NdhL, NdhN, NdhO, NdhS, NdhV in the S02:Δ*flv3* strain at all sampling times (Figs. 4 and 8). After careful analysis of the location of the last-mentioned subunits in cryo-EM structures of the complexes [64], they appeared to be involved in Fd binding to the NDH-1 complex and electron transfer to the PQ pool. The second group was composed of NdhH, NdhJ, NdhK, NdhM, which slightly increased in abundance in the S02:Δ*flv3* only on d9 of the experiment. The remaining subunits NdhA, NdhB, NdhE, NdhG were at the same level in both strains throughout the sucrose production experiment. In addition, an NDH-1 M core complex assembly factor (NDH-1 M AF), chlororespiratory reduction 6 (Crr6) protein [9] showed increased abundance in S02:Δ*flv3* compared to S02 at all sampling points.

#### Differential expression of proteins involved in carbon metabolism

The S02:Δ*flv3* showed prominent upregulation of three enzymes from low glycolysis, including phosphoglycerate kinase (Pgk), and pyruvate kinase (Pk) all through the sucrose production experiment and also 2,3-bisphosphoglycerate-independent phosphoglycerate mutase (PGAM) on d1.5 (Fig. 4). The positive regulator Rre37 (Sll1330) involved in the control of glycolysis and OPP [55] showed decreased abundance on d1.5 in S02:Δ*flv3*, but clear upregulation in the subsequent time points. The negative regulator Sll1334 that inhibits this pathway [39] was not upregulated at any point. The phosphoketolase (PKET) pathway was another metabolic route with differential protein expression, evidenced by high abundance of phosphoketolase enzyme (PKET1) in S02:Δ*flv3* strain, compared to S02, at all analyzed sampling times. Furthermore, the citric acid cycle (TCA) enzymes, such as aconiate hydratase (AcnB) and malate dehydrogenase (Mdh) decreased in abundance in S02:Δ*flv3* compared to the S02 strain in consecutive sampling times. Importantly, phosphoenolpyruvate carboxylase (PEPC) responsible for CO_2_ assimilation, increased in abundance in S02:Δ*flv3* from d5 to d9. After the pyruvate node, the enzymes PhaB, PhaC and PhaE, involved in the PHB synthesis, were upregulated on d9 in S02:Δ*flv3*. Also, the phasin PhaP enzyme, controlling the size of PHB granules, changed in S02:Δ*flv3* from low abundance on d1.5 to highly abundant on d5 and even more on d9 (Fig. 4, Supporting Fig. 3). From enzymes involved in carbohydrates metabolism glycogen debranching enzyme GlgX was enhanced in this strain towards the end of the cultivation period.

While there were no notable differences in the levels of the overexpression target Sps between S02:Δ*flv3* and S02, the subsequent enzyme in sucrose pathway, sucrose phosphate phosphatase (Spp) showed downregulation in S02:Δ*flv3* strain on d5-d9. Notably, the response regulator Slr1588 (Rre39) that natively controls the transcription of Sps [5], was upregulated towards the end of the experiment. This, however, did not have any effect on Sps overexpression because the artificial construct lacks the target sequences in the promoter region [56]. In addition, phosphoglucomutase (PGM2/PMM; slr1334), the enzyme reversibly converting G1P to G6P [10, 40], was upregulated in S02:Δ*flv3* on d9 (Fig. 4).

#### Differential accumulation of plasma membrane transporters

**Fig. 9:**
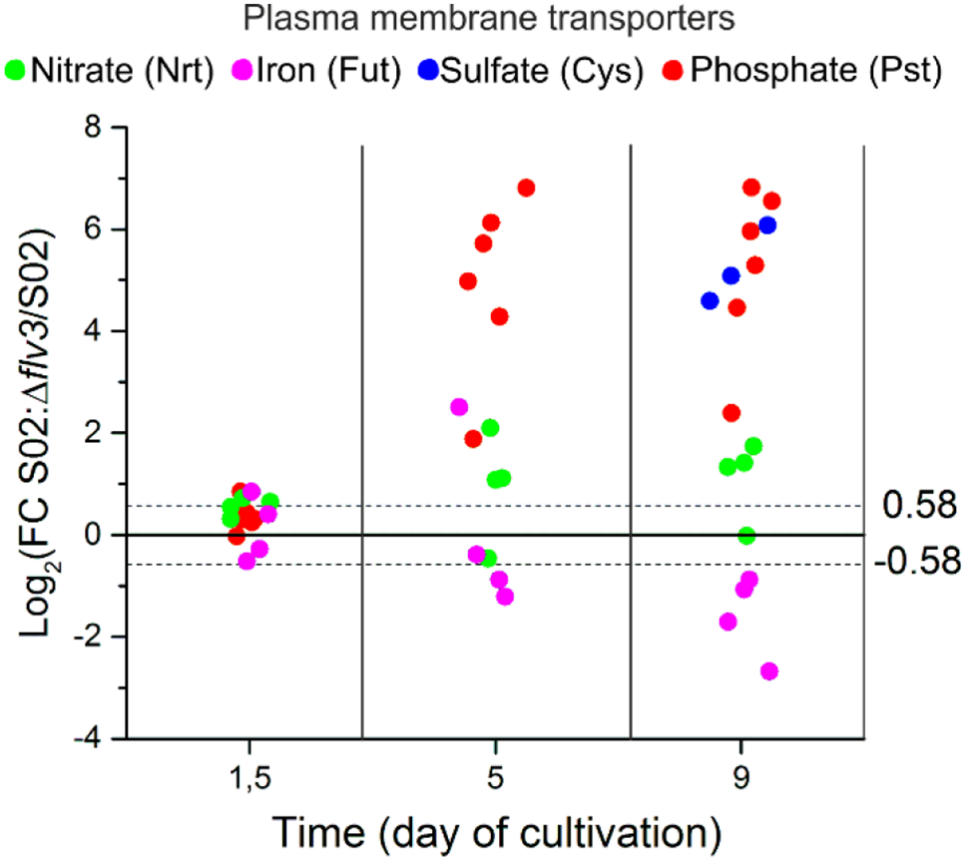
Differential expression of plasma membrane transporters. The values are expressed as log_2_FC of protein abundance in strain S02:Δ*flv3* compared to S02. The data presented are statistically significant with p-value ≤ 0.05. The practical threshold for data interpretation was set at -0.58 ≥ log_2_FC ≥ 0.58

Phosphate transporters (the Pst family) were induced in S02:Δ*flv3* strain on d5 and d9 (Figs. 4 and 9). Similarly, the sulfate transporters (CysA/W) accumulated to a high level on d9 in the same strain. Coordinated induction of potassium ion transporters such as KdpB was also observed in S02:Δ*flv3* compared to S02. Potassium compensates for negative charges of phosphate groups in polyphosphate (PP) granules always present in mixotrophic cultures. Nitrate acquisition via ABC-type nitrate and nitrite bispecific transporter (NRT) system increased gradually from d1.5 till d9. Periplasmic iron-deficiency-induced protein A (IdiA) was highly induced in S02:Δ*flv3* at d5 but diverted this trend by d9 when a strong decrease in the abundance of IdiA was detected. Other iron transport proteins (FutA/C) showed lower abundances in S02:Δ*flv3* strain compared to S02.

#### Differential expression of other maintenance proteins

The proteins involved in the biosynthesis of iron sulfur clusters (SufR/B/C/D/S) were induced in S02:Δ*flv3* after 9 days of growth in the sucrose production experiment. At the same time, the iron-sulfur cluster carrier (MRP) was downregulated at all sampling points (Fig. 4). The two isoforms of glutamate-amonia ligase (Gln) involved in GS-GOGAT pathway, which is the main pathway to assimilate nitrogen to the carbon skeleton of 2-oxoglutaric acid (2-OG), decreased in abundance in S02:Δ*flv3* compared to S02 at all sampling times of the experiment for GlnA but only on d9 in case of GlnN. Several other proteins indicated important alteration in the S02:Δ*flv3* strain metabolism. These include an increase in 2-isopropylmalate synthase (LeuA) of leucin biosynthetic pathway on d1.5, the coordinated upregulation of periplasmic phosphate binding protein (SphX) and polyphosphate kinase (Ppk), responsible for synthesis of polyphosphate chains (Fig. 4) on d5 and d9. Nitrate reductase (NirA and NarB) also increased in abundance in S02:Δ*flv3* compared to S02 on d5 and d9. One of the most downregulated proteins in S02:Δ*flv3* from dataset was a circadian clock protein KaiC3.

## Changes in key metabolites in S02:Δ flv3 compared to S02 in the course of growth and sucrose production

**Fig. 10:**
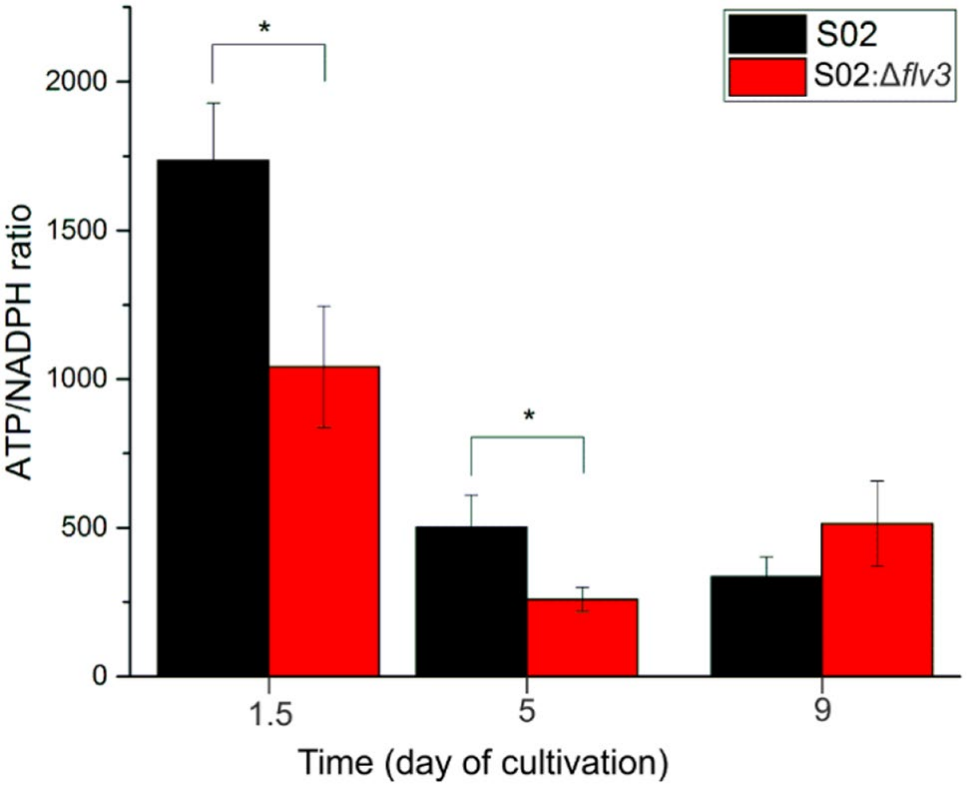
The ATP/NADPH ratio calculated for S02 and S02:Δ*flv3.* The values were calculated based on the ATP and NADPH abundances acquired in the LC/MS analysis performed on the extracts from S02 and S02:Δ*flv3*. Means and standard deviations were calculated from 3–4 independent measurements. Asterix indicates statisticaly significant difference with p-value < 0.05

We next set to assess whether the observed proteomic changes in S02:Δ*flv3*, in comparison to S02, correlate with the changes in the main metabolites of the respective strains over the course of the sucrose production for 9 days. Seventy metabolites were targeted for quantification from the whole cell extracts of the S02:Δ*flv3* and S02 *Synechocystis* strains at d1.5, d5 and d9 after initiating the sucrose production experiments. Out of the 70 targeted metabolites, 53 compounds were detected and quantified with relative quantification methodology (Table 1; Fig. 4). The most striking difference between the two strains was the accumulation of acetic acid (AcAc) and lactic acid (LacAc) as well as glutamic acid (Glu) and arginine (Arg) in the S02:Δ*flv3* strain on d5 and d9 in comparison to S02. In addition, glucose (Glc) derivatives such as 6-phospho-gluconate (6PG) and the pool of glucose-1-phosphate (G1P) and fructose-6-phosphate (F6P) also increased in abundance specifically in the S02:Δ*flv3* strain. An opposite trend occurred in photorespiratory metabolites, such as 2-phosphoglycolate (2-PG), which decreased in S02:Δ*flv3* in comparison to S02. Intermediate compounds from the low glycolytic pathway such as 2-phosphoglycerate (2PG) and phosphoenolpyruvate (PEP), as well as several amino acids such as lysine (Lys), serine (Ser) and glycine (Gly) decreased in abundance in S02:Δ*flv3* in comparison to S02. The abundances of alanine (Ala) and ornithine (Orn), on the contrary, increased in abundance in S02:Δ*flv3* compared to S02, particularly by the end of the sucrose production experiments. In addition, the ATP/NADPH ratio was calculated based on data registered with LC/MS method (Fig. 10) for evaluating overall energetic status of the cell. The ATP/NADPH ratio in S02:Δ*flv3* is significantly lower than in S02 strain on d1.5 and d5 d5 but evened out by the end of the nine-day cultivation.

**Table 1:** Differential abundance of metabolites represented with log_2_FC in S02:Δ*flv3* strain compared to S02 at consecutive sampling times

Metabolite	S02Δflv3 vs. S02 d1.5	S02Δflv3 vs. S02 d5	S02Δflv3 vs. S02 d9
Compound name	Difference (log_2_FC)	Difference (log_2_FC)	Difference (log_2_FC)
2-OG	-0.8	-0.1	**1.5**
2PG	**-1.1**	**-1.0**	0.2
2-PG	**-1.4**	-0.8	-0.7
6PG	**-1.4**	**0.8**	**0.9**
AcAc	-0.5	**2.4**	**2.4**
Ala	-0.2	0.6	**0.9**
AMP	0.0	-0.1	**-0.8**
Arg	0.1	**0.6**	**1.5**
Asp	-0.1	**0.5**	**1.1**
ATP	0.0	**-1.2**	0.1
CitAc	0.2	**0.9**	**1.9**
diOHAcetone-P	-1.9	**2.4**	0.5
F6P	**-1.1**	**1.1**	**1.4**
Fum	0.2	**-0.5**	**-0.6**
G1P	**-1.1**	**1.1**	**1.4**
G6P	**-1.3**	**0.9**	**1.3**
Gln	-0.1	**-0.8**	0.2
Glu	**0.6**	**0.7**	**1.4**
Gly	**-0.4**	-0.2	**-0.6**
GMP	0.2	0.1	**-0.8**
GSSG	**-0.5**	**-0.4**	**-3.4**
LacAc	**-1.3**	**1.2**	**1.5**
Lys	**-2.3**	**-2.2**	-0.4
Met	**-0.4**	-0.1	**-0.9**
NADP^+^	**0.8**	**-0.4**	**-0.9**
Orn	**-0.4**	0.3	**1.9**
PEP	**-0.7**	**-1.7**	**-1.2**
Pro	**-0.9**	0.2	**0.7**
Pyr	**-1.1**	-0.3	**1.4**
Ser	**-1.8**	-0.3	0.1
Trp	**1.0**	**0.4**	-0.1
UDP	**0.4**	**-0.7**	**-0.9**
UMP	0.0	0.2	**-0.9**
UTP	**0.5**	**-2.2**	-1.1

The bolded numbers represent statistically significant difference with p value < 0.05. The practical threshold for data interpretation was set to -0.58≥ log_2_FC ≥ 0.58. Abbreviations: Gln–glutamine, Asp–aspartic acid, ATP- adenosine triphosphate, UMP-uridine monophosphate, AMP-adenosine monophosphate, Arg-arginine, Ala-alanine, 2-OG-2-oxoglutarate, Fum-fumrate, CitAc-citric acid, diOHAcetone-P-dihydroxyacetone phosphate, GMP-guanosine monophosphate, AcAc – acetic acid, Pyr-pyruvate, Met-methionine, Glu-glutamate, Orn-ornitine, Gly-glycine, UTP-uridine triphosphate, G1P-glucose-1-phosphate, F6P- fructose-6-phosphate, 6PG-6-phosphogluconate, G6P-glucose-6-phosphate, GSSG-glutathion oxidized, Pro-proline, UDP-uridine diphosphate, LacAc-lactic acid, 2-PG-2-phosphoglycolate, PEP-phosphoenolpyruvate, Trp-tryptophan, 2PG-2-phosphoglycerate, NADP^+^-nicotinamide adenine dinucleotide phosphate, Lys-lysine, Ser-serine

### Accumulation of cellular storage compounds

**Fig. 11:**
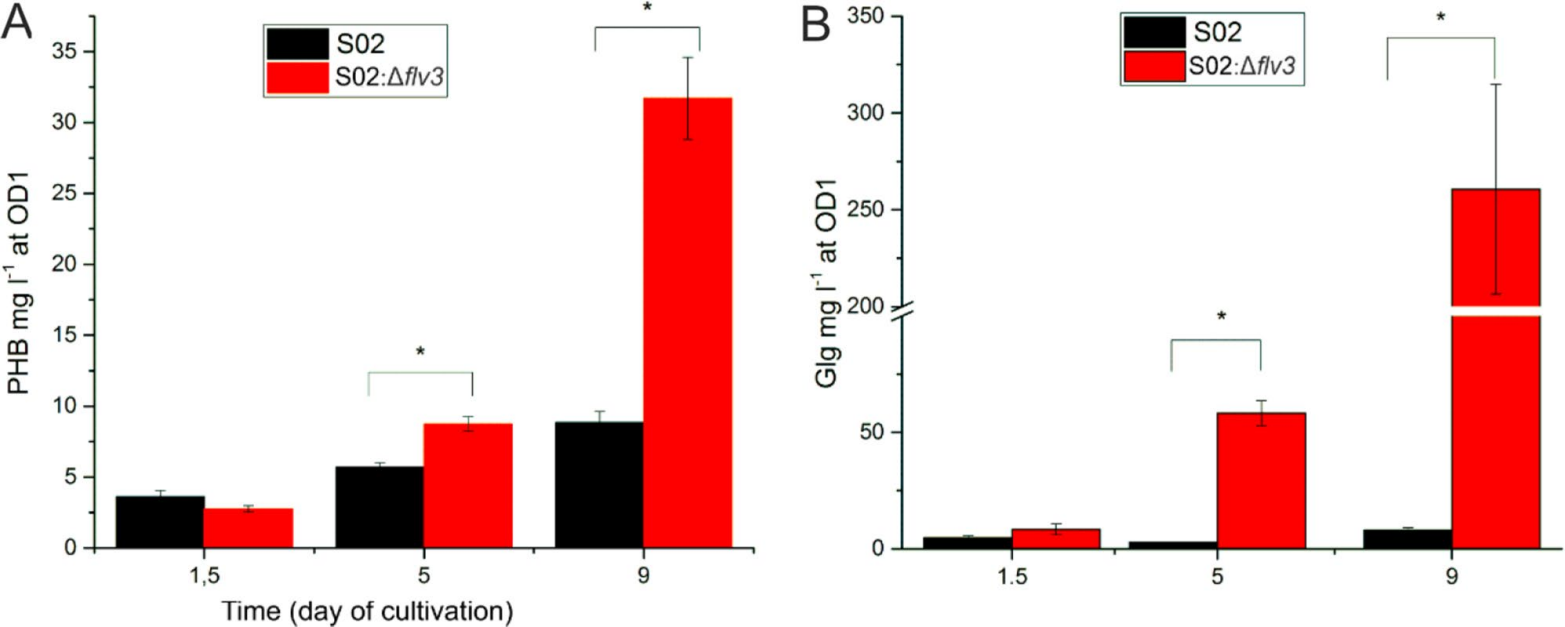
Accumulation of the polymers PHB and GLK in S02 and S02:Δ*flv3* cells. PHB (**A**) and GLG (**B**) (both represented as mg per liter of culture at OD1) accumulation in the S02 (black) and S02:Δ*flv3* (red) cells at the sampling times of day 1.5, 5 and 9. Means and standard deviations were calculated based on four independent cell cultures (*n* = 4)

To follow the carbon allocation in cells during the sucrose production experiment, the two strains S02:Δ*flv3* and S02, were analyzed with respect to the accumulation of storage polymers glycogen (GLK) and polyhydroxybutarate (PHB) (Figs. 4 and 11). These storage compounds were quantitated from four independent replicates of each strain at sampling times of d1.5, d5 and d9. Starting from similar initial levels GLK and PHB in d1.5, the strain S02:Δ*flv3* accumulated a significant amount of both polymers by d9 in comparison to the S02 strain. The amount of PHB increased by over three-fold in S02:Δ*flv3* and the corresponding accumulation of GLK was nearly 30-fold.

## Discussion

### Deletion of *flv3* induces a change in growth mode during sucrose production

**Fig. 12:**
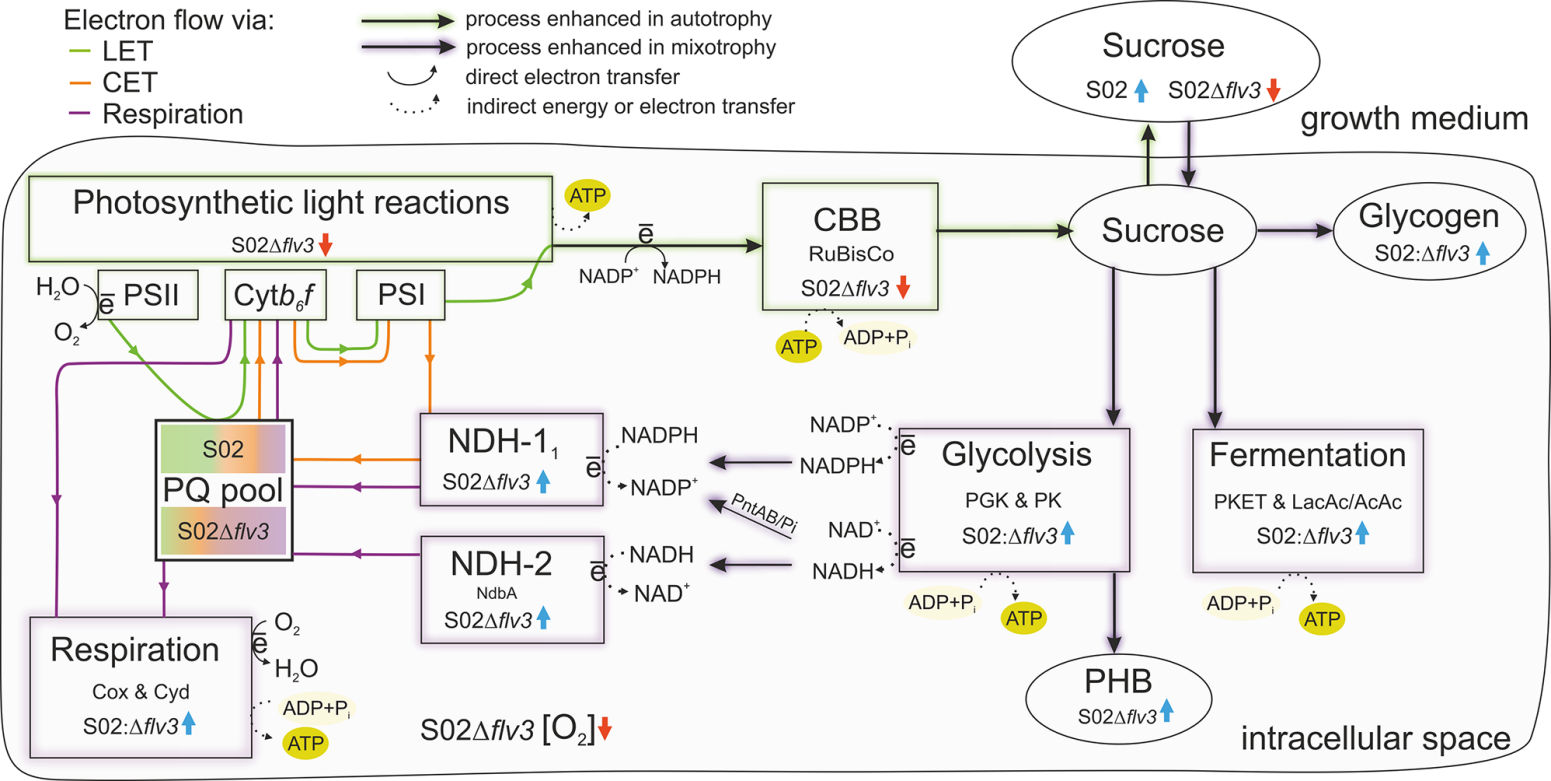
Summary scheme of rearrangements in bioenergetic and metabolic pathways of the *Synechocystis* S02:Δ*flv3* strain compared to S02. Initially (day 1.5), both strains maintain autotrophic growth (green shadow) where all the sucrose produced is secreted into the external growth medium. Over time, S02:Δ*flv3* starts to consume sucrose from the growth medium and adapts to mixotrophic growth (purple shadow), where PHB and glycogen become indirect carbon sinks. This effect is due to the absence of the Flv electron valve from PSI in S02:Δ*flv3*, and leads to a rearrangement of electron fluxes favoring the respiratory chain (purple) over photosynthesis, including LET (green) and CET (orange). Disruption of LET leads to decrease in S02:Δ*flv3* energetic status, which further results in CBB down-regulation and increased energy sourcing from glycolytic and fermentation pathways. Reducing equivalents provided by carbohydrate degradation fuel the respiratory electron flux via NDH-1 and NDH-2 to the PQ pool, which is located at the intersection of both photosynthetic and respiratory electron transport pathways. The ratios of electron fluxes through the PQ pool are derived from rearrangements of metabolic and bioenergetic pathways in the presence or absence of Flv3 protein over the course of the sucrose production experiment. Blue arrow indicates up-regulation and red arrow indicates down-regulation of the process, and accumulation or decrease in the amount of the metabolite, respectively

This study focused on the cascade of bioenergetic and metabolic changes induced by deletion of the Flv3 protein, which accepts electrons from PSI, in the engineered sucrose-producing *Synechocystis* S02 strain [56]. To allow comparison between Flv3 deletion strain S02:Δ*flv3* and the reference strain S02 [56], the cells were cultivated under 200 µmol photons m^− 2^s^­1^ continuous light, 1% CO_2_, and salt stress to induce sucrose production. During the first two days of cultivation, the strains cannot be distinguished on the basis of cell growth (Fig. 2A), sucrose accumulation in the medium (Fig. 2B), or the intracellular levels of the storage compounds, PHB (Fig. 11A) and glycogen (Fig. 11B). Thereafter, the phenotypic behavior of the strains markedly diverges: while S02 continues autotrophic growth and maintains steady production of sucrose, S02:Δ*flv3* starts to effectively consume sucrose from the growth medium and reverts to mixotrophy (Fig. 2B). This experimental setup provided a platform to assess the metabolic shift resulting from the absence of Flv3 in *Synechocystis* and allowed us to visualize the transition to mixotrophy at the proteome and metabolome levels over the nine-day cultivation period (Fig. 4). The results reveal the extent of the underlying metabolic and bioenergetic rearrangements and their molecular interactions that are directly or indirectly linked to the functions of Flv3 in the whole cell context (Fig. 12).

Absence of Flv3 in S02:Δ*flv3* results in shutdown of the Flv1/Flv3-mediated electron valve, which protects the photosynthetic machinery by transferring excess of electrons from PSI to O_2_. This Flv1/Flv3 pathway is the main native pathway for O_2_ photoreduction in *Synechocystis* under high CO_2_ concentrations [47], and a sink for a significant proportion of water-derived electrons particularly under high light [1]. The parallel Flv2/Flv4 pathway is not expressed under these conditions (Fig. 4; [47, 65]. and therefore cannot compensate for the lack of Flv1/Flv3 in S02:Δ*flv3* under our experimental conditions. The extra electrons accumulated in the LET of the S02:Δ*flv3* strain can potentially be redirected to enhance carbon assimilation (e.g. under low light conditions) in the presence of an efficient downstream sink [56]. However, the source-sink balance is highly dependent on cultivation conditions and the metabolic context as well as the overall energy status of the cell. In the conditions applied here, the cellular energy status of S02:Δ*flv3* is lower as compared to S02 as determined by decrease in the intracellular ATP/NADPH ratio in S02:Δ*flv3* (Fig. 10). Therefore, the redox balance is unfavorable to support efficient CO_2_ fixation, leading to activation of glycolytic metabolism to produce more ATP – as opposed to producing more sucrose. These effects take place before any phenotypic changes become apparent.

### Induction of glycolytic metabolism in S02:Δ*flv3* at the onset of mixotrophy

Sucrose consumption in S02:Δ*flv3* is accompanied by increased respiration (Fig. 12; Thiel et al [56]. , and proteomic changes (Fig. 4) characteristic for wild type *Synechocystis* grown in the presence of glucose [36]. The first indication is the up-regulation of 3-phosphoglycerate mutase (PGAM), an enzyme common to all glycolytic pathways [41], which catalyzes the conversion of 3PG to 2PG. In parallel, PKET1, the key enzyme in the phosphoketolase pathway that converts F6P to acetyl phosphate, is up-regulated (Fig. 4). While contributing to the flux towards acetyl-CoA, PKET1 is also known to effectively down-regulate photosynthetic CO_2_ fixation by competing with CBB when the cellular ATP level is low [33]. The bidirectional phosphoglycerate kinase (PGK) is also clearly upregulated in S02:Δ*flv3* throughout the 9-day experiment, and is expected to contribute specifically to the glycolytic flux towards 3GP (Fig. 4). Other highly up-regulated enzymes include pyruvate kinase (PK), which catalyzes the final step of glycolysis [18, 28], and phosphoenolpyruvate carboxylase (PEPC) [48], which fixes CO_2_ to replenish the TCA cycle via oxaloacetate, and also increases the glycolytic flux from PEP to pyruvate [45].

Under mixotrophic conditions, the anabolic and catabolic reactions take place simultaneously in a single compartment, and are tightly co-regulated to sustain balanced growth of the cell. The breakdown of carbohydrates is natively controlled via a complex signaling network that involves the sigma factor SigE, response regulator Rre37, and Hik8-signal transduction cascade [2, 39]. In the obtained data, we only see the upregulation of Rre37, that activates gene expression related to sugar catabolism, and correlates well with the increased abundance of PK in S02:Δ*flv3*. Typically Rre37 is induced under nitrogen starvation, and has been linked with the accumulation of glycogen and 2-OG [23] that we observe in the ∆*flv3* background. In this context it is noteworthy that many regulatory proteins are allosterically controlled by other proteins or small molecules, and consequently, the observed abundance does not necessarily correlate with the biological efficiency.

At metabolite level, sucrose consumption is accompanied by a gradual increase in the sucrose-derived metabolites G6P, G1P, F6P and 6PG (Fig. 4; Table 1). The different sugar intermediates are interconvertible, and their fate is regulated by the energetic status and redox balance of the cell. F6P is the substrate for the PKET pathway, which is upregulated in S02:Δ*flv3*, while G6P is most likely shared between glycolysis and glycogen biosynthesis. At the lower levels of metabolism, the associated changes in S02:Δ*flv3* include a gradual increase in TCA cycle intermediates (CitAc and 2OG) and derived amino acids (Glu, Arg, Asp). Despite the use of carbohydrate substrates, the levels of the glycolytic pathway intermediates (2PG, PEP, PYR) are reduced in the S02:Δ*flv3* strain, but this may simply reflect a rapid turnover of transient metabolites, as the consequence of increased flux towards the TCA cycle in S02:Δ*flv3* (Fig. 4).

### The S02 and S02:Δ*flv3* strains use different strategies to maintain optimal growth

At the beginning of the 9-day experiment the engineered sucrose pathway serves as a photosynthetic electron sink in both S02 and S02:Δ*flv3* strains. While S02 maintains stable photosynthetic sucrose production until reaching a steady state growth after day 7, the production in S02:Δ*flv3* slows down already after day 2, and the strain starts to consume sucrose from the medium (Fig. 2B). This observation clearly shows that solely the presence of a carbohydrate substrate in the growth medium is not sufficient to induce mixotrophic growth in S02 when the photosynthetic reactions and the electron sink efficiency are in equilibrium. In the absence of Flv3 source-sink balance is disturbed, and the cell is unable to maintain efficient photosynthesis using engineered sucrose biosynthetic pathway as a sink. In S02:Δ*flv3* the induction of glycolytic metabolism is accompanied by an increased accumulation of glycogen and PHB, which now serve as the indirect photosynthetic electron sinks. The same phenomenon has been previously reported also in engineered sucrose-importing *Synechococcus elongatus* PCC 7943 [52], where sucrose uptake directly leads to glycogen buildup. Characteristically, the two storage compounds in cyanobacteria are closely coupled, and the balance between glycogen accumulation and consumption is linked to PHB biosynthesis under specific conditions [29].

It is difficult to monitor sucrose biosynthesis in S02:Δ*flv3* during mixotrophic growth when sucrose is being consumed by the cells, but expectedly the process is at least partially inhibited. At the proteome level we observe downregulation of Spp in the sucrose pathway in this strain, as well as the accumulation of the response regulator Rre39, that natively controls the transcription of Sps responsible for the preceding limiting step [5, 53]. It is noteworthy however, that Rre39 cannot block Sps expression from the plasmid-based overexpression construct present in the target strains, as the gene is under the transcriptional control of a promoter that does not contain the Rre39 operator sequences [56].

Sucrose utilization leads to increased flux through central carbon metabolic pathways and accumulation of 2OG, indicative of a carbon-rich (high C/N) state in S02:Δ*flv3*. As the C/N ratio is tightly regulated in cyanobacteria [13], the increase in carbon availability in S02:Δ*flv3* activates mechanisms for nitrogen assimilation that are also observed in WT *Synechocystis* grown under mixotrophic conditions (presence of Glc) [36]. Such changes recorded in S02:Δ*flv3* include up-regulation of proteins involved in nitrate transport (NrtABCD) and assimilation (NarB), and increased levels of the amino acids glutamine (Glu), arginine (Arg) and the intermediate orinitine (Orn) (Fig. 4; Table 1), which are typical indicators of ammonia up-shift in *Synechocystis* [4, 12].

### Flv3 deletion induces interconversions between NDH-1 complexes, favoring respiratory electron flux over the CET pathway

The proteomic data revealed striking changes in the composition of the S02:Δ*flv3* NDH-1 complexes, which have multiple functions in cyanobacteria. All the four NDH-1 complexes are composed of a common NDH-1 M core of 17 subunits, complemented by different isoforms of the functionally relevant NdhD and NdhF membrane subunits. NDH-1_1_ (NdhD1/NdhF1) and NDH-1_2_ (NdhD2/NdhF1) function in respiration and CET, whereas NDH-1_3_ (NdhD3/NdhF3) and NDH-1_4_ (NdhD4/NdhF4) function in carbon concentration and CET [43, 66]. While differential abundance of functionally relevant subunits indicates abundance of the corresponding NDH-1 complex, the consequences of non-stoichiometric changes in the NDH-1 M core subunits, as observed in the presente experimental setup are not yet fully understood. In our current proteomic data, 14 out of the 17 NDH-1 M core complex subunits were detected and divided into three groups based on differential abundance in S02:Δ*flv3* and S02 (Figs. 4 and 8). The first group of proteins, up-regulated 3-fold in S02:Δ*flv3* compared to S02 strain from d1.5 to d9, includes the hydrophilic arm proteins of the NDH-1 M core complex involved in electron transfer from ferredoxin (Fd) to the PQ pool. These include the regulatory oxygenic photosynthesis specific (OPS) subunits (NdhL/N/O/S/V) [34] and iron cluster containing NdhI. Recently, it has been shown that NdhO, NdhV and NdhI form a cavity for Fd binding and allow electron transfer through the hydrophilic arm, while NdhL is located in the complex adjacent to the electron acceptor PQ cleft [64]. All the above OPS subunits are necessary for electron transfer, while NdhO was additionally found to negatively modulate CET [69]. Furthermore, the high accumulation of the NDH-1 assembly factor Crr6 protein, which is involved in the ligation of the FeS clusters to NdhI [9], was observed in S02:Δ*flv3* strain throughout the experiment, in contrast to S02. In the second group of proteins, the NDH-1 M subunits NdhH, NdhJ, NdhK and NdhM show a 50% up-regulation in S02:Δ*flv3* only on d9. These four subunits form a subcomplex, an assembly intermediate, in the cytoplasm and are essential for all three NDH-1 pathways: CET, CCM and respiration [19]. The third group of proteins include the NDH-1 M membrane-embedded proteins NdhA, NdhB, NdhE, NdhG, which do not differ in abundance between S02:Δ*flv3* and S02. Non-stoichiometric changes in the NDH-1 M core subunits have previously been reported in the differential accumulation of the NdhI and NdhK subunits under high light conditions [64], suggesting a possible regulatory role as a valve for excess electrons from the LET.

Differential expression of the NDH-1M core subunits and the NdhD/NdhF isoforms provided a means to assess the interconversion of NDH-1 complexes in S02:Δ*flv3* compared to S02 and to estimate their function in CET, respiration or CCM. Similar abundances of the Ndh core membrane-embedded subunits (group three) provide evidence that the total content of functional NDH-1 complexes does not differ between S02:Δ*flv3* and S02 during the nine days of the sucrose production experiment. From the downregulation of NdhF3 and NdhF4 at d1.5, we conclude that NDH-1_3_ and NDH-1_4_ start to be degraded in the S02:Δ*flv3* strain as compared to S02, reflecting the decrease in CO_2_ assimilation (Fig. 4). At the same time, the assembly of the NDH-1_1_ complex is enhanced, as evidenced by the accumulation of NdhD1 protein on d9, which is likely to promote either respiration or CET. Although the specific activity of the NDH-1_1_ complex in respiration or CET is difficult to determine, the abundances of distinct subunits of NDH-1 have been connected to one function over the other. In our data, we observed up-regulation of NdhO in S02:Δ*flv3* suggesting that respiratory path of NDH-1_1_ is favored over CET in S02:Δ*flv3* compared to S02. Another mechanism of CET regulation relies on the formation of the NDH1-CpcL-PBS-PSI trimer supercomplex, which allows for more efficient electron transfer between complexes [15, 16]. The CpcL linker protein of this supercomplex is highly abundant in S02:Δ*flv3* at d1.5, but decreases dramatically by d9 compared to S02 (Figs. 4 and 7). This protein is crucial for anchoring the CpcL-PBS complex in the thylakoid membrane [31], allowing efficient energy transfer from PBS to PSI. The reduction of CpcL in S02:Δ*flv3* indicates a lowered ability to form the supercomplex and confirms the reduction of NDH-1_1_ function in CET and the enforcement of its function in respiration.

The mechanism of CET relies on electron transfer from PSI via the NDH-1_1_ complex to the PQ pool with simultaneous transfer of 4 protons across the thylakoid membrane to lumen, thus efficiently contributing to PMF [16]. Acidification of thylakoid lumen results in more efficient ATP production through the ATPase. Impaired CET in S02:Δ*flv3* thus weakens the photosynthetic ATP production compared to S02. At the same time, the upregulation of glycolytic metabolism occurs in S02:Δ*flv3*, which in the presence of O_2_ is linked to the respiratory activity of NDH-1_1_ to produce ATP from NAD(P)H released in glycolysis.

### Respiratory proteins and pathways are generally strengthened in S02:Δ*flv3*

In cyanobacteria some components of the thylakoid electron transport chain(s), particularly the PQ pool and the Cyt*b*_*6*_*f* complex, participate in both photosynthesis and respiration. During the sucrose production, several changes in the protein levels of respiratory pathways reflect the metabolic transition of S02:Δ*flv3* from photoautotrophy to mixotrophy. In addition to the changes in NDH-1 described above, these include the up-regulation of NdbA, which is also involved in electron transfer to the PQ pool, and the RTOs Cyd and Cox, which ultimately transfer the electrons from PQ to O_2_ during respiration.

In parallel to the down-regulation of photosynthesis, the proteomic data suggests that an increased proportion of electrons transferred to the PQ pool by NDH-1_1_ in S02:Δ*flv3* is likely to be directed to respiration (Figs. 4 and 12). This may be a mechanism to relieve the electron pressure in PSI in the absence of Flv3, that protects the cells under high light and another route for electron transfer from via PQ pool to molecular oxygen. In addition to the accumulation of NDH-1_1_ that accepts electrons from NADPH (via ferredoxin), the plastoquinone reductase NDH-2 (NdbA) transfers electrons from NADH to PQ pool was upregulated in S02:Δ*flv3*. NdbA, known to be natively accumulated in *Synechocystis* under mixotrophic and heterotrophic conditions [22], notably increased in abundance in S02:Δ*flv3* during sucrose consumption. These results support the view that the respiratory flux during mixotrophy is maintained in a cooperative manner by NDH-1_1_ and NdbA, which together allow the transfer of electrons from photosynthetic water splitting and carbohydrate degradation to the PQ pool and further to O_2_ via Cyd and Cox (Fig. 12).

The two main pathways discussed above that funnel electrons to the PQ pool are further linked with other mechanisms that contribute to the respiratory flux in *Synechocystis*. One of these involves pyruvate: ferredoxin oxidoreductase (PFOR), which catalyzes the interconversion of pyruvate and acetyl-CoA with the concomitant reduction of Fd. The electrons from Fd can then be transferred to NDH-1_1,_ thereby providing an efficient route for sugar-derived electrons to the PQ pool during mixotrophic growth [62]. In addition, the pyridine nucleotide transhydrogenase (PntAB) catalyzes electron transfer between NADH and NADPH under mixotrophic conditions in *Synechocystis* [25], from where the electrons can be further transferred to Fd by the small ferredoxin: NADP^+^-oxidoreductase isoform FNR_S_ [3]. Although no changes in PFOR or PntAB protein levels were observed, they may still play an important role in regulating the glycolytic electron flux in S02:Δ*flv3* during mixotrophic growth. The electrons derived from glycolytic processes are directed to the respiratory electron transport, which is coupled to proton translocation across the thylakoid membrane, contributing to PMF and further used for ATP synthesis.

### Increased respiration leads to transient O_2_ limitation in S02:Δ*flv3*

The up-regulation of respiratory metabolism (Figs. 4 and 12) is bound to lead to a dramatic increase in oxygen consumption, which, together with reduced photosynthetic O_2_ production [56], leads to transient oxygen limitation and induction of anaerobic glycolytic metabolism in S02:Δ*flv3*. At the metabolite level this is evidenced by the accumulation of the fermentative products acetate (AcAc) and lactate (LacAc), which are typically produced in *Synechocystis* under anoxic glycolytic conditions [42]. Oxygen limitation is also supported by the increased levels of NADH during the course of sucrose consumption, as the regeneration of NAD^+^ via the respiratory chain can be severely limited in the absence of sufficient O_2_ [62]. Notably, such anaerobic conditions would lead to a profoundly reduced ATP yield as compared to fully aerobic glycolysis, which together with the downregulation of photosynthetic ATP production, could explain the temporary decrease in ATP levels on 5d in S02:Δ*flv3* (Figs. 4 and 10; Table 1). However, the overall energy status of the S02:Δ*flv3* strain appears to be improved towards the end of the 9-day cultivation (Fig. 10). In addition to improved growth in comparison to S02, this is reflected in higher accumulation of glycogen and PHB with up-regulation of the respective key enzymes as well as phosphate transporters and polyphosphate granule forming proteins in S02:Δ*flv3* (Figs. 4 and 11).

## Conclusions

The engineered *Synechocystis* S02:Δ*flv3* strain is unable to sustain photosynthetic sucrose production under constant high light and high CO_2_ due to the absence of the flavodiiron protein Flv3. Although *Synechocystis* does not have direct alternative photosynthetic mechanisms to compensate for the lack of Flv3, the cells re-establish the metabolic balance by optimizing the use of the available resources, sucrose, CO_2_ and light, by switching to mixotrophy. While enhanced glycolytic metabolism provides energy for the S02:Δ*flv3* cell, the down-regulation of photosynthesis is accompanied by (i) increased capacity to direct electrons to the respiratory chain for O_2_ reduction and (ii) accumulation and interconversion of inert storage molecules that act as indirect photosynthetic electron sinks. This demonstrates the immense flexibility of cyanobacterial metabolism to survive under changing bioenergetic and metabolic conditions and highlights the importance of understanding the interactions between different electron transfer pathways and carbon sinks when engineering efficient cyanobacterial cells for biotechnological use.

### Electronic supplementary material

Below is the link to the electronic supplementary material.

Supplementary Material 1

Supplementary Material 2

## Data Availability

The metabolomic LC-MS measurement raw data and mz/ml files are available in GNPS-MassIVE data repository with 10.25345/C51834D4P. The proteomic datasets generated and/or analysed during the current study are available in the Panorama Public data repository associated to ProteomeXchange Consortium under a link https://panoramaweb.org/S02dflv3_timecourse.url
