# Supplementary material for: Interplay between photosynthetic electron flux and organic carbon sinks in sucrose-excreting Synechocystis sp. PCC 6803 revealed by omics approaches
Source: Microb Cell Fact. 2024 Jul 1;23:188. doi: 10.1186/s12934-024-02462-6 (PMC11218172; doi:10.1186/s12934-024-02462-6)
Supplement: Supplementary file 2 — Supplementary Material 2 [file 12934_2024_2462_MOESM2_ESM.docx]

__

**Supporting Figure 1 Representation of statistical parameters of the proteomic dataset.**

Values of statistical significance were calculated in MSStats Shiny software to ensure the appropriate practical threshold for data interpretation. With 4 replicates used in the experiment with FDR 0.05 we reach the statistical power of 0.9 for fold change of 1.5 (FC≥1.5).

_
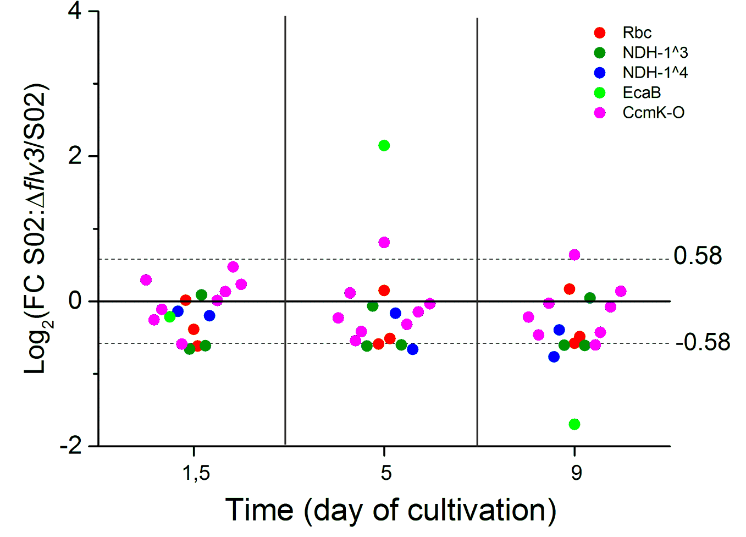
_

**Supporting Figure 2** **Differential expression of carbon concentrating mechanism (CCM) protein components.**

The values are expressed as log_2_FC of protein abundance in strain S02:Δ*flv3* compared to S02. The data presented are statistically significant with p-value ≤ 0.05. The practical threshold for data interpretation was set at -0.58 ≥ log_2_ FC ≥ 0.58.

Abreviation; Rbc- RuBisCO, EcaB – carbonic anhydrase, Ccm – carbon concentrating proteins (components of csrboxysomes)


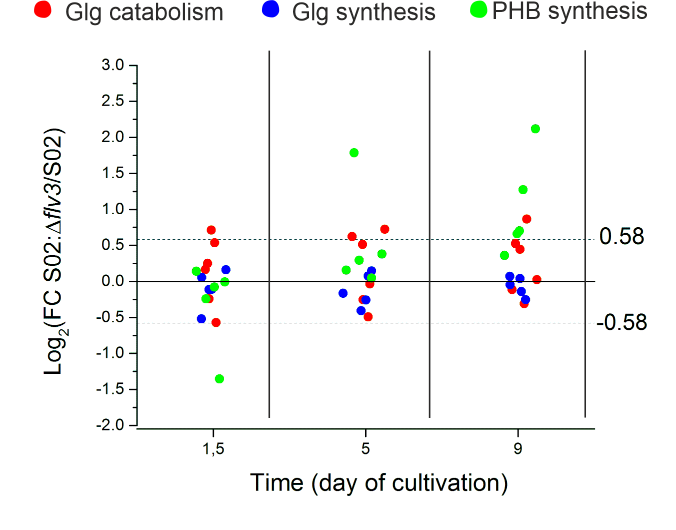


**Supporting Figure 3 Differential expression of enzymes involved in glycogen (Glg) metabolism and PHB biosynthesis protein components.**

The values are expressed as log_2_FC of protein abundance in strain S02:Δ*flv3* compared to S02. The data presented are statistically significant with p-value ≤ 0.05. The practical threshold for data interpretation was set at -0.58 ≥ log_2_ FC ≥ 0.58.

**Supporting Figure 4 ATP/ADP ratio calculated for S02 and S02:Δ*flv3*.**

The values were calculated based on the ATP and NADPH abundances registered in the LC/MS analysis performed on the extracts from S02 and S02:Δ*flv3*. Means and standard deviations were calculated from 3-4 independent measurements. Asterix indicates statisticaly significant difference with p-value <0.05.
